# Educational intervention on biosafety with hospital hygiene and cleaning workers*

**DOI:** 10.1590/1518-8345.7449.4518

**Published:** 2025-05-19

**Authors:** Ana Carolina Sobota Vasconcelos, Maria Alice Santos Lobo, Claudia Aparecida Godoy Rocha, Renan Sallazar Ferreira Pereira, Leidiene Ferreira Santos, Mirian Cristina dos Santos Almeida

**Affiliations:** 1 Universidade Federal do Tocantins, Campus Palmas, Palmas, TO, Brazil; 2 Universidade Federal de São João Del-Rei, Campus Centro-Oeste Dona Lindu, Divinópolis, MG, Brazil

**Keywords:** Continuing Education, Occupational Health, Hospital Housekeeping, Personal Protective Equipment, Occupational Exposure, Inservice Training

## Abstract

to evaluate the effectiveness of an educational intervention with hospital cleaning and sanitizing workers in protecting against injuries caused by biological agents.

this was a prospective, quasi-experimental, before-and-after study with a single group, analyzing workers’ behavior regarding adherence to standard precautions. Data was collected from 106 cleaning and sanitizing workers through a demographic and occupational questionnaire and a knowledge and behavioral survey on preventing diseases caused by biological agents. Participants’ behavior related to biosafety was observed before and after the educational intervention. Descriptive and inferential statistical analysis.

77.4% of the workers are female and approximately 42% have worked for up to a year. As for the level of education, 39.6% had completed high school and 29.2% had incomplete primary education. The median score for adherence to standard precautions: pre-intervention 14 points and post-intervention 17 points. Among the observations before dressing, adherence to hand hygiene with soap and water rose from 41.7% to 75.0% after the intervention.

the educational intervention, based on theoretical-practical workshops and active methodologies, improved adherence to biosafety practices, as evidenced by both reported behavior and direct observation. The leadership and commitment of hospital managers are crucial for the continuity of education and biosafety practices, ensuring the safety of professionals and patients. Future studies should focus on the health of hospital cleaning and sanitizing workers, including educational programs and the relationship between their activities and patient safety against infections in healthcare services.

## Highlights

**(1)** Including hospital hygiene and cleaning professionals in biosafety. **(2)** Continuing education to promote biosafety among cleaning workers. **(3)** Continuing education using active methodologies. **(4)** Continuing education to promote adherence to PPE among hospital cleaning workers.

## Introduction

Biosafety is defined as actions aimed at preventing, controlling, reducing, or eliminating risks arising from activities that could compromise the quality of life, human health, and the environment^(1)^. Risks are classified as physical, biological, chemical, ergonomic, or accident-related, which are means of contamination in both research laboratories and health units^(2)^.

Pathogenic microorganisms represent a significant concern in closed environments, especially in services such as hospitals, due to the risk of causing hospital-acquired infections^(3)^. Therefore, professionals working in these environments are more susceptible to infectious diseases due to frequent exposure to viruses, bacteria, and fungi during their work^(4)^.

From this perspective, the importance of the use of Personal Protective Equipment (PPE) by hospital employees is noteworthy, considering the Ministry of Labor’s Regulatory Standard (RS) No. 6, which states that the use of PPE is “any device or product, for individual use, used by the worker, intended to protect against occupational health and safety risks”^(5)^.

It should be noted that hospital cleaning and sanitizing workers handle potential infectious agents daily, which can become a major source of contamination if not managed correctly^(6)^. Despite global recognition of the importance of cleaning in healthcare, cleaning workers remain undervalued and are often ignored by clinical staff on busy wards^(7)^.

Likewise, implementing health education approaches for cleaning agents can improve their knowledge of biosafety, thus contributing to more effective infection control in healthcare environments^(8)^.

To this end, it highlights the importance of Permanent Health Education (PHE) in the construction of learning in the daily life of the work team. PHE encompasses educational activities in health, management, and social control, to critically analyze users’ needs and promote changes in the reality of health work^(9-9)^. Strengthening the safety culture within healthcare facilities and providing continuous training are essential components of PHE, contributing to the protection of workers^(11)^.

Given this context, there is a clear need for studies that address PHE with hospital cleaning and sanitizing workers, with a focus on biosafety. Thus, the guiding questions arise: “What is the effectiveness of an educational intervention in the adherence to biosafety measures by hospital cleaning and sanitizing workers in protecting against injuries caused by biological agents?” and “Can PHE activities contribute to protecting these workers from injuries caused by biological agents?”.

The contributions of this study to the hospital sector are manifold. Firstly, by highlighting the importance of educational interventions in adherence to biosafety practices, the study could provide a safer environment for both health professionals and patients. In addition, continuous and specific training for cleaning and sanitizing workers could reduce the incidence of hospital-acquired infections, improving the quality of care and clinical outcomes. Finally, by promoting awareness and appropriate training, it is hoped that this study will contribute to building an organizational culture focused on safety and risk prevention and strengthening institutional occupational health and safety policies.

Therefore, this study aimed to evaluate the effectiveness of an educational intervention with hospital cleaning and sanitizing workers in protecting against injuries caused by biological agents.

## Method

### Study design

This is a prospective, quasi-experimental, before-and-after, single-group study analyzing workers’ behavior in terms of adherence to standard precautions to protect against injuries caused by biological agents. The writing of this study was guided by the Strengthening the Reporting of Observational Studies in Epidemiology (Standards for Quality Improvement Reporting Excellence) (SQUIRE) checklist^(12)^.

### Research scenario

The study was carried out in a large health unit located in the state of Tocantins, Brazil. The institution provides 100% of its services through the Unified Health System (UHS) and is characterized as a general, public, tertiary-level teaching hospital. It was selected because it is a teaching hospital and a place where teams of cleaning and sanitizing workers work in environments with a high risk of infection by biological material. The sectors that were part of this research were: adult emergency room, surgical center, and admissions.

### Population, selection criteria, and sample definition

All hospital cleaning and sanitizing workers working in the institution’s units were invited to take part in the study. The inclusion criteria were: being hospital cleaning and sanitizing workers linked to the hospital being researched, not being on sick leave or in a managerial position. Those who were on leave for any reason during the data collection period and those who did not take part in the educational intervention were excluded.

For the observation stages, the eligibility criteria used were workers who had taken part in the other stages of the survey. To calculate the sample size, the population was 123 workers, with a 99% confidence interval and a 5% sampling error, resulting in a minimum sample of 104 workers. Possible losses due to refusals or absences were taken into account, and all workers present in the workplace were invited to participate in the study. In the pre-intervention 115 workers took part by answering the questionnaires; in the educational intervention, 106 workers took part, as well as in the post-intervention.

Thus, 106 workers participated in all stages, making up the final sample of workers who participated in both the pre-intervention, intervention, and post-intervention stages.

For the researchers’ observation of adherence to biosafety measures in the workplace by hospital cleaning and sanitizing workers, both in the pre-intervention and post-intervention stages, 12 workers were observed. This simple random sample represented around 10% of the total number of participants in the first stage of the research.

### Instruments used

The data collection instrument consisted of a questionnaire made up of three parts: (1) demographic and occupational; (2) knowledge and behavior regarding the prevention of diseases caused by biological agents, and (3) questions about the types of PPE used for precautions based on transmission.

The demographic and occupational questionnaire was constructed by the authors of this study, containing the following items: biological sex, age, schooling, work shift and working time, and previous participation in training on PPE (pre-intervention).

The questionnaire on knowledge and behavior regarding the prevention of injuries caused by biological agents (pre- and post-intervention) was adapted from the Brazilian version of the Compliance With Standard Precautions Scale (CSPS-PB)^(13)^, validated through methodological processes such as translation, consensus between judges, back-translation, semantic validation, and pre-test.

The CSPS scale represents alternatives for measuring the adherence of nursing professionals to infection control practices. Due to the lack of specific instruments for hospital cleaning and sanitizing workers and to adapt to their work activity, the questionnaire adapted from the CSPS was kept at 20 items. Of these, items 3, 6, 7, 12, 13, 14, 15, 16 and 17 remained unchanged from the original. Items 1, 8, 10 and 19 were modified, while items 2, 4, 5, 9, 11, 18 and 20 were removed. In addition, seven new items relating to the use of masks, dressing, and undressing of PPE were added. Previously, a pre-test was carried out with some hospital cleaning and sanitizing workers at the institution where the study took place.

Following the same assumptions as the CSPS-PB, the questionnaire had four answer options that indicated the frequency of compliance with the standard precautions (SP), comprising “always”, “often”, “rarely” or “never”. Since professionals were expected to fully adhere to the SP, it was expected that the “always” option would account for the majority of responses^(13)^.

The SP compliance score ranges from zero to 20, and the closer it is to 20, the better the professionals’ adherence. For the answer option “always”, a score of “one” was assigned, and for the options “often”, “rarely” or “never” the score assigned was “zero”.

To record the observation of the participants’ behavior (pre- and post-intervention), we used a form called the Checklist for assessing adherence to measures to prevent injuries caused by biological agents/use of PPE. This instrument is an adapted version of the PPE Checklist for dressing and undressing^(14-14)^.

### Data collection

Data collection took place between December 2022 and April 2023 by previously trained researchers and was carried out in four stages, distributed during the pre-intervention, intervention, and post-intervention.

In the first stage, a meeting was held with the workers from each shift to present the study, invite them, and administer the questionnaire on their demographic and occupational profile, and their knowledge and behavior concerning the prevention of injuries caused by biological agents.

The completed questionnaires were placed by the participants themselves in an unmarked, sealed envelope and handed to the researchers when they had finished filling them in. During data collection, the researchers were available to answer any questions about filling in the questionnaire.

The second stage consisted of observing the behavior of the participants (pre- and post-intervention) to assess adherence to measures to prevent injuries caused by biological agents/use of PPE. It took place over six field days, totaling 72 hours, and it was possible to carry it out on all shifts.

The different dynamics and routines of the service were observed, at alternating times and periods, taking into account the possibilities presented by the field. This approach was fundamental for clarity, identification, and understanding of the organization and dynamics of the dressing and undressing process in the setting studied.

The observers avoided establishing direct contact with the participants so as not to influence their behavior. When observing the participants and recording their behavior, each worker was observed at alternate times by two researchers^(16)^.

In the third stage of data collection, the educational intervention was implemented. To this end, a teaching plan was drawn up according to the need for continuing education identified through the questionnaires applied and participant observation.

This stage was carried out in the form of a theoretical-practical workshop, using active methodologies, with the following themes: hand hygiene technique; biological risks and routes of contamination; sequence indicated for dressing and undressing; types of precautions and; PPE according to the type of precaution.

As part of the active methodologies, we used hand hygiene practice in the “Box of Truth”; the “Simulation of the risk of contamination” theater, where we explained the transmissibility of biological agents; a dressing and undressing workshop and the “Game of Errors”, using images and simulations of work situations where the worker pointed out errors relating to the use of PPE.

In the fourth stage, one month after the educational intervention, a post-test was carried out using the questionnaire on knowledge and behavior about the prevention of diseases caused by biological agents and observation of the post-intervention participants.

### Educational intervention

The educational intervention with hospital cleaning and sanitizing workers was developed after identifying previous knowledge and observing practices related to biosafety.

We opted for the format of theoretical-practical workshops, using dynamic moments to assimilate the content explained. The activities carried out were:

*Simulation of hand hygiene in the “Box of Truth”* - This consists of an organizing box with a side opening for inserting hands and an opening in the lid for viewing. A five-watt black light was incorporated into the inner right-hand side. To enhance the illumination from the black light, the inside of the box was coated in black.

As a sanitizing method, a 70% alcohol gel solution was used, added to a transparent dye that becomes fluorescent in the presence of black light. This solution was made available to professionals for hand hygiene.

After the application, the professionals were taken to the “Box of Truth” to check the coverage of their hands with the fluorescent alcohol solution, identifying the presence of areas that had not been properly sanitized.

*Theatrical play* - During the demonstration by the member of the research team, a simulation of the transmission of biological agents was presented. The scene depicted a professional collecting contaminated garbage, with the garbage bag previously smeared with gouache paint to represent the aforementioned biological agents.

In the role-play, after collecting the garbage, the professional opens a door and touches his mask, scratching his nose with the gloves that had handled the contaminated garbage bag. Afterward, when removing the gloves, the professional omits to sanitize his hands, greets a co-worker with a handshake and continues his work activities.

*Occupational safety* - This dynamic involved the use of two balloons, both inflated with air, only one of which was wrapped in transparent adhesive tape. It was explained that the balloon without tape represented the professional who doesn’t use PPE or does so inappropriately, while the balloon with tape represented the professionals who use PPE correctly.

A needle symbolized an accident at work and the risks of contamination. When the needle was inserted into the balloon without the tape, it broke easily, while when it was inserted into the balloon with the tape, the balloon remained intact.

*Game of errors* - Images or simulations of work situations were shown which highlighted mistakes in the use of PPE, presenting the risks of contamination, as well as inadequacies in dressing and undressing practices. The workers identified the errors in the scenes and pointed out the correct actions for each situation.

*What’s missing?* - Actors (researchers) were shown dressed in PPE, as indicated by all the precautions. The workers, divided into groups, observed and verbally identified which PPE was missing.

The pedagogical workshop was designed on the basis of meaningful learning, a multimodal approach, and the application of active methodologies. It included discussions on the topics presented, as well as moments dedicated to practical activities, with the aim of providing a more in-depth understanding of workers in the hospital cleaning and sanitizing area.

### Data analysis

Descriptive analysis was carried out using absolute and relative frequencies for the qualitative variables, and means and standard deviations for the quantitative ones. McNemar’s test was used to compare the items that make up the SP at pre- and post-intervention, and the score obtained by the adherence score was compared using the Wilcoxon test, as the data did not adhere to the normal distribution, which in turn was assessed using the Shapiro-Wilk test. The significance level was 5%. The program used was Stata (StataCorp, LC) version 15.0.

The observation records made by the two researchers had a kappa coefficient of 0.94, which showed a high degree of agreement.

### Ethical aspects

This study was carried out under Resolution 466/12 of the National Health Council (CNS)^(17)^. The project obtained institutional authorization for data collection and was assessed and approved by the Research Ethics Committee (REC) of the Federal University of Tocantins (UFT) (CAAE-63028922.2.0000.5519/Opinion 5.694.479).

## Results

The average age of the study participants was 40.9 years (SD 11.9), with a minimum age of 21 and a maximum of 67. Table 1 shows the demographic and occupational profile of the 106 hospital cleaning and sanitizing workers.

**Table 1 -:** Numerical and percentage distribution of the demographic and occupational profile of hospital cleaning and sanitizing workers (n = 106). Tocantins, TO, Brazil, 2022-2023

Variables	n (%)
**Biological sex**	
Female	82 (77.4)
Male	24 (22.6)
**Education**	
Elementary school complete	10 (9.4)
Elementary incomplete	31 (29.2)
High school complete	42 (39.6)
High school incomplete	21 (19.8)
Higher education complete	1 (0.9)
Higher education incomplete	1 (0.9)
**Work shift**	
Daytime	63 (59.4)
Evening	43 (40.6)
**Working time (years)**	
Up to 1	40 (37.8)
1 to < 2	27 (25.4)
2 to < 3	7 (6.6)
3 or more	32 (30.2)
Previous participation in PPE training*	
Yes	95 (89.6)
No	11 (10.4)
**PPE Theoretical/Practical**	
Theoretical	75 (70.7)
Practical	26 (24.6)
Both	5 (4.7)
How many times have you attended PPE training*	
01-2	63 (59.4)
03-4	(19.9)
5+	22 (20.7)

*Personal Protective Equipment

Within the spectrum of biological sex, there is a marked female predominance, i.e. 77.4%. In terms of education, around 40% of professionals have completed secondary school, and around a third have not completed elementary school. A mere fraction, 1.8%, went on to higher education. Notably, 59.4% of those interviewed worked the day shift.

When considering length of service, approximately 42% have worked for up to one year, with a quarter having worked for between one and two years. The remainder work for between two and three years, or exceed the three-year mark.

At the heart of professional training is PPE training. With regard to this variable, 89.6% had already undergone previous training. However, 70.7% are theoretical, although around a quarter have had practical or theoretical-practical training.

Table 2 illustrates the percentage of adherence before and after the educational intervention for each item on the Adherence to Standard Precautions questionnaire.

**Table 2 -:** Adherence to Standard Precautions at pre- and post-intervention by hospital cleaning and sanitizing workers (n = 106). Tocantins, TO, Brazil, 2022-2023

**Questions**	**Pre-intervention**	**Post-intervention**	p*
1. I wash my hands when I touch contaminated handles and equipment.	72 (67.9%)	106 (100.0%)	<0.001
2. I use alcohol-based hand sanitizer as an alternative if my hands are not visibly dirty.	69 (65.1%)	96 (90.6%)	<0.001
3. The sharps container is only disposed of when it is full.	96 (90.6%)	106 (100.0%)	0.002
4. I remove PPE † in a designated place.	59 (55.7%)	86 (81.1%)	<0.001
5. I shower and change into private clothing in the event of large spills, even if I have worn PPE†.	63 (59.4%)	83 (78.3%)	0.002
6. I wear long rubber gloves when I am exposed to body fluids, blood or blood products, and any patient excretions.	52 (49.1%)	0 (0%)	<0.001
7. I sanitize my hands immediately after removing gloves.	92 (86.8%)	106 (100.0%)	<0.001
8. I wear a surgical mask in combination with goggles and an apron whenever there is a possibility of splashes or spills.	40 (37.7%)	75 (70.8%)	<0.001
9. My mouth and nose are covered when I wear a mask.	95 (89.6%)	106 (100.0%)	0.001
10. I reuse a surgical mask or disposable PPE ^†^ .	71 (67.0%)	106 (100.0%)	<0.001
11. I wear an apron/cap when I am exposed to blood, body fluids, or any patient excretions.	68 (64.2%)	106 (100.0%)	<0.001
12. I dispose of material contaminated with blood, body fluids, secretions, and patient excretions in white plastic bags, regardless of the patient’s infectious status.	98 (92.5%)	106 (100.0%)	0.008
13. I wear procedure gloves to decontaminate visibly dirty equipment.	94 (88.7%)	106 (100.0%)	<0.001
14. I avoid touching my eyes, nose, and mouth with unwashed/sanitized hands.	93 (87.7%)	106 (100.0%)	<0.001
15. I avoid touching surfaces (e.g. furniture, door handles, and healthcare equipment) with contaminated gloves or other PPE ^†^ or with contaminated hands.	78 (73.6%)	106 (100.0%)	<0.001
16. I avoid touching the front of the mask and when I do, I immediately sanitize my hands.	67 (63.2%)	106 (100.0%)	<0.001
17. I remove the mask using the appropriate technique (i.e. I don’t touch the front of the mask, but remove it by the straps).	74 (69.8%)	106 (100.0%)	<0.001
18 I sanitize my hands after removing the mask.	86 (81.1%)	106 (100.0%)	<0.001
19. After use, I dispose of disposable PPE ^†^ in the infectious waste garbage can (white bag).	86 (81.1%)	106 (100.0%)	<0.001
20. After use, I remove the apron and try to hold onto the inside of it to avoid contaminating myself.	75 (70.8%)	106 (100.0%)	<0.001

*McNemar p = p-value for comparison between pre- and post-educational intervention; ^†^Personal Protective Equipment

Initially, it should be noted that when comparing the behavior reported before and after the educational intervention, all the items showed a positive, statistically significant difference, with a value of p<0.001. In contrast to item 7, where “I wear rubber gloves when I am exposed to body fluids, blood or blood derivatives and any excretion from patients” became zero after the intervention, due to the absence of PPE in the service (replaced by latex gloves).

**Figure 1 -:**
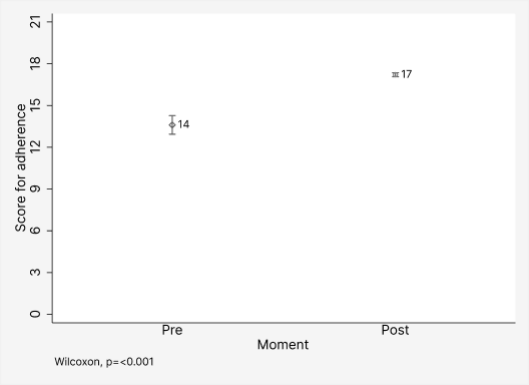
Effects of the educational intervention on the score of adherence to standard precautions by hospital cleaning and sanitizing workers (n = 106). Tocantins, TO, Brazil, 2023

The median overall SP adherence score, compared between pre- and post-intervention, was higher in the post-intervention with 17 points (Figure 1).

Table 3 shows adherence to specific PPE for respiratory precautions before and after the intervention.

**Table 3 -:** Adherence to PPE* for specific respiratory precautions before and after the intervention by hospital cleaning and sanitizing workers (n = 106). Tocantins, TO, Brazil, 2023

Variables	Pre				Post			
	**Always**	**Sometimes**	**Rarely**	**Never**	**Always**	**Sometimes**	**Rarely**	**Never**
***Respiratory transmission - aerosols***								
Cap	99 (93.4%)	3 (2.8%)		4 (3.8%)	106 (100.0%)			
Surgical mask	24 (22.6%)	1 (0.9%)	4 (3.8%)	77 (72.7%)				106 (100.0%)
Long-sleeved apron (not waterproof)	80 (75.5%)	10 (9.4%)	7 (6.6%)	9 (8.5%)	106 (100.0%)			
Procedure gloves	93 (87.8%)	7 (6.6%)	1 (0.9%)	5 (4.7%)	106 (100.0%)			
N95 or PFF2 ^†^ mask	88 (83.0%)	14 (13.2%)		4 (3.8%)	106 (100.0%)			
Goggles or face shield	31 (29.2%)	13 (12.3%)	9 (8.5%)	53 (50.0%)				106 (100.0%)
Waterproof long-sleeved apron	33 (31.1%)	11 (10.4%)	9 (8.5%)	53 (50.0%)				106 (100.0%)
N95 seal test, PFF2 ^†^ or similar	65 (61.3%)	12 (11.3%)	8 (7.6%)	21 (19.8%)	84 (79.3%)	15 (14.1%)	2 (1.9%)	5 (4.7%)
***Respiratory droplet transmission***								
Surgical mask	80 (75.5%)	6 (5.7%)	2 (1.8%)	18 (17.0%)	106 (100.0%)			
Long-sleeved apron (not waterproof)	80 (75.5%)	15 (14.2%)	1 (0.9%)	10 (9.4%)	106 (100.0%)			
Procedure gloves	93 (87.7%)	9 (8.5%)	1 (0.9%)	3 (2.9%)	106 (100.0%)			
Goggles or face shield	32 (30.2%)	13 (12.3%)	8 (7.5%)	53 (50.0%)				106 (100.0%)
Waterproof long-sleeved apron	36 (34.0%)	6 (5.7%)	8 (7.5%)	56 (52.8%)				106 (100.0%)
Standard cloth or canvas apron	19 (17.9%)	9 (8.5%)	8 (7.6%)	70 (66.0%)				106 (100.0%)

*Personal Protective Equipment; ^†^Class 2 filtering face piece

The simple descriptive analysis shows an increase in the percentage of adherence to various PPE, both for aerosol and droplet precautions, with emphasis on items such as caps, long-sleeved aprons (not waterproof), procedure gloves, and N95 masks or Class 2 Filtering Facepieces (PFF2), with adherence becoming 100% “always”.

In the area of respiratory transmission by aerosols, adherence to the continuous use of caps, long-sleeved aprons (not impermeable), procedure gloves, N95 masks, or FFP2 after the intervention was 100%. Before the intervention, the use of surgical masks was low, with only 22.6% of professionals always using them, and 72.6% never using them. However, in the post-intervention period, the use of this PPE fell to zero, which was the expected result, given that the protocol is not to use surgical masks for aerosol respiratory transmission precautions.

It can be seen that, in relation to the N95, FFP2, or similar seal test, there was an increase in adherence from 61.3% in the pre-intervention period to 79.3% in the post-intervention period.

With regard to respiratory transmission by droplets, 75.5% of professionals always wore surgical masks and long-sleeved aprons (not impermeable) at the time of the pre-intervention.

Post-intervention, this rate reached 100%. Procedural gloves were frequently worn by 87.7% of the interviewees before the intervention, a figure which also rose to 100% afterward. On the other hand, before the intervention, 30.2% of the professionals always wore goggles or face shields and 34.0% constantly wore a long-sleeved waterproof apron.

After the intervention, both practices were completely discontinued. The standard cloth or canvas apron was used by only 17.9% pre-intervention, and as the other PPE mentioned, its use also ceased post-intervention.

Table 4 shows the items observed by the researchers prior to dressing, during dressing and when cleaning and sanitizing workers were undressed, when they randomly observed the behavior of 12 professionals before and after the intervention, in relation to biosafety measures.

**Table 4 -:** Procedures observed before and during dressing and undressing for standard precautions before and after the educational intervention by hospital cleaning and sanitizing workers (n = 12). Tocantins, TO, 2022-2023

Before dressing	Pre-intervention	Post-intervention
Sanitizes hands with soap and water	5 (41.7%)	9 (75.0%)
Separates and validates the integrity of the PPE*	11 (91.7%)	12 (100.0%)
***Apron*** : fastens neck and waist ties	7 (58.3%)	12 (100.0%)
**During dressing**		
***Surgical mask***		
Wears a surgical mask	11 (91.7%)	12 (100.0%)
**During dressing**	Pre-intervention	Post-intervention
Places the nose clip on the back of the nose	11 (91.7%)	12 (100.0%)
Positions the elastics behind the ears or tie the ribbons	11 (91.7%)	12 (100.0%)
Ensures that the mask covers the nose, mouth, and chin	7 (58.3%)	10 (83.3%)
***Safety Glasses***		
Does not wear	12 (100,0%)	12 (100,0%)
***Face shield***		
Does not wear	12 (100.0%)	12 (100.0%)
***Puts on procedural gloves*** covering the cuffs of the apron sleeves	12 (100.0%)	12 (100.0%)
Puts on the ***Cap*** covering your hair and ears	11 (91.7%)	12 (100.0%)
***Long rubber boots***		
Does not wear	12 (100.0%)	12 (100.0%)
**During undressing**		
***Procedure gloves***		
Holds the cuff of the glove on the non-dominant hand and remove the first glove	8 (66.7%)	12 (100.0%)
Places the index finger under the cuff of the glove and removes the second glove	1 (8.3%)	12 (100.0%)
No	11 (91.7%)	0 (0.0%)
Disposes of gloves in infectious waste	12 (100.0%)	12 (100.0%)
***Sanitizes hands*** with 70% alcohol preparation	6 (50.0%)	12 (100.0%)
***Apron***		
Removes the necktie first and then the waist tie	5 (41.7%)	11 (91.7%)
Places your index finger on the inside of the cuff and pull the apron over your non-dominant hand.	2 (16.7%)	8 (66.7%)
With the hand protected by the apron sleeve, holds the other sleeve while sliding and removing the arm and dominant hand from the apron.	1 (8.3%)	10 (83.3%)
Grasps the inside of the apron (near the shoulder) and removes the sleeve from the non-dominant arm	1 (8.3%)	10 (83.3%)
Continues to turn the apron inside out, folding it into a bundle	1 (8.3%)	10 (83.3%)
Discards the apron in the infectious waste garbage can	9 (75.0%)	12 (100.0%)
***Sanitize hands*** with 70% alcohol preparation	6 (50.0%)	12 (100.0%)
***Glasses or protection***		
Does not wear	12 (100.0%)	12 (100.0%)
***Was wearing a hat***	11 (91.7%)	12 (100.0%)
Places the index fingers inside the cap next to the ears and slide the cap upwards, backward, and sideways	8 (66.7%)	12 (100.0%)
Disposes of the cap in infectious waste	11 (91.7%)	12 (100.0%)
***Sanitize hands*** with 70% alcohol preparation	1 (8.3%)	10 (83.3%)
***Surgical mask***		
Was wearing	9 (75.0%)	12 (100.0%)
Holds the elastics behind the ears, or undo the ribbon loops at the back of the head	9 (75.0%)	12 (100.0%)
Pulls the mask forward, holding onto the elastic bands or ribbons, without touching its surface	8 (66.6%)	12 (100.0%)
Disposes of the mask in infectious waste	9 (75.0%)	12 (100.0%)
Sanitizes hands with 70% alcohol preparation	0 (0.0%)	9 (75.0%)
***Long-cut waterproof rubber boot***		
He/She wasn’t using	12 (100.0%)	12 (100.0%)

*Personal Protective Equipment

It can be seen that all the items evaluated “before dressing” had an increase in adherence after the educational intervention.

During dressing, all the procedures associated with the surgical mask, including its use, positioning, and adjustment, showed 100% adherence after the intervention, with the exception of the procedure of ensuring that the mask covers the nose, mouth, and chin, which increased from 58.3% to 83.3%. In contrast, the use of the cap, which covers hair and ears, achieved 100% adherence after the intervention.

As for the procedures observed during breastfeeding, there was an increase in adherence to many practices. In the case of glove removal, the technique of holding the cuff of the glove in the non-dominant hand to remove the first glove, and placing the index finger under the cuff of the glove to remove the second, both achieved 100% adherence after the intervention.

Hand hygiene with 70% alcohol after removing the apron and surgical mask went from 50.0% and 0.0% to 100.0% and 75.0%, respectively. The procedure of removing the apron, turning it inside out, and folding it, increased from 8.3% to 83.3%.

## Discussion

This study focused on analyzing the demographic and occupational profile of hospital cleaning and sanitizing workers at a hospital in the state of Tocantins, Brazil, in 2022/2023, as well as the effectiveness of the ongoing biosafety continuing education intervention.

In line with previous studies, a female predominance was identified in hospital cleaning and sanitizing^(18)^.

In terms of educational background, a detailed analysis revealed critical nuances. The limited presence of professionals with higher education resonated with the literature, which suggests that these roles are commonly occupied by individuals with fewer years of academic training^(19)^.

The equitable distribution between day and night shifts can affect both the well-being of the professionals and the operational efficiency of the institutions. Hospitals operate 24 hours a day, requiring cleaning professionals to be prepared to work shifts that cover the entire period, both day and night. Maintaining hospital hygiene is essential throughout the day, but working at night can present additional health challenges^(20)^. Work shifts for older or inexperienced staff should be organized in short periods during the afternoon to minimize the risk of accidents at work. In addition, older staff should be allocated to low-risk units to prevent accidents^(21)^.

The data highlighted a high level of participation in previous PPE training, most of which was theoretical. This dichotomy in training, together with the frequency of PPE updates, raises questions about the effectiveness and quality of the training offered. The lack of adequate training was also perceived in other departments, where participants said they did not have all the information they needed to carry out their tasks^(22)^.

This reinforces the importance of implementing effective training in health units based on workers’ needs. This approach provides a better chance of learning, resulting in greater adherence by professionals to individual protection measures, while at the same time reducing vulnerability to risks^(23)^. On the other hand, knowledge alone does not necessarily indicate a behavior change, since this can be related to intrinsic and extrinsic factors.

In this sense, health literacy is linked to the acquisition of knowledge and the transformation of individual behaviors. It can be conceptualized as a set of fundamental skills and contextual resources that people need to acquire, understand, evaluate, communicate, and apply information and services in a variety of ways, in various scenarios throughout their lives, to improve their health and well-being^(24)^.

Health education has the potential to promote changes in human behavior. It can encourage reflection on the organization of cleaning work, identify the factors that cause illness, and discuss strategies to reduce them. This helps re-signify self-care practices and improves the ability to cope with the problems that affect the physical and mental health of workers in the Hospital Cleaning Service^(25)^.

In this way, skills that go beyond mere knowledge and training play a significant role in the development of practical skills, such as dressing, undressing, and hand hygiene, as well as in the formation of positive attitudes. This highlights the beneficial impact of the intervention carried out through theoretical and practical workshops using active methodologies.

After the educational intervention, there was a significant increase in adherence to standard precautions. These results are in line with existing literature which identifies the effectiveness of various strategies, including training, feedback, and facilitating systems^(26-26)^.

There was a significant increase in the use of alcohol as a sanitizing agent when hands were not visibly dirty, showing a knowledge gap combined with a lack of daily practice. Hand hygiene, whether with water and liquid soap or alcoholic preparations, per the five moments established for this practice in health services, should be a routine already internalized by all health professionals. It is imperative to reiterate its importance as a fundamental measure for the prevention and control of infections, both now and in the future^(28)^. Therefore, healthcare facilities must provide ongoing training aimed at compliance with the hand hygiene standards established by the competent bodies^(29-29)^.

The connection between the implementation of biosafety practices and the preservation of workers’ health and patient safety is a vital one. This emphasizes that biosafety is a functional and operational procedure of extreme relevance in the various health services, and should be recognized as an instrument of protection for both the patient and the professionals involved in providing health care^(31)^.

There were notable improvements in the following aspects: identification of the correct place to remove PPE, proper use of PPE when dealing with bodily fluids, understanding of the ideal combinations of PPE to ensure the safety of both the professional and the patient, consideration of the reuse of PPE, use of PPE for the decontamination of equipment and surfaces, and correct adoption of measures to avoid contamination during the de-briefing process. This improvement is in line with previous studies that have emphasized the protection provided by such equipment in hospital environments^(32)^.

According to the guidelines of Brazil’s National Health Surveillance Agency (ANVISA), it is recommended that hygiene and cleaning professionals in hospital services wear glasses or a face shield (especially when there is a risk of splashing organic or chemical materials), an apron (if there is a risk of contact with patient fluids or secretions that could go beyond the barrier of the contact apron, the professional should wear an impermeable apron), long rubber gloves and long impermeable boots^(28)^.

As for the disposal of PPE and hospital materials, all the professionals demonstrated full adherence to items 3, 12, and 19 in the self-reported knowledge questionnaire. This corroborates other studies, where improper disposal of materials was identified as one of the main causes of accidents involving biological material, occurring more frequently when professionals were not wearing PPE, accounting for 51% of cases^(33)^.

Adherence to some PPE practices before the intervention was modest. This low pre-intervention adherence suggested that there were gaps in awareness or understanding of the importance of PPE. In undressing, there was a considerable increase in adherence to the correct procedures post-intervention, in line with the guidelines that stress the importance of this process to avoid contamination^(34)^.

The study is justified by the scarcity of scientific studies focusing on the population of hospital cleaning and sanitizing workers. Among the limitations found, we highlight the lack of validated questionnaires that address specific biosafety measures for these workers. The standard precaution questionnaire does not mention the appropriate PPE for this category, such as rubber gloves and impermeable long rubber boots. It is therefore suggested that studies be carried out to validate biosafety questionnaires aimed at this population.

Another limitation of the study is the application of the results obtained. Observing the participants may interfere with their behavior concerning adherence to biosafety measures, since they may perceive that they are being observed at that moment, which may alter their actions.

The implications for professionals are significant, as the study highlights the need to develop and validate specific tools to assess adherence to biosafety practices among hospital cleaning and sanitizing workers. In addition, it reinforces the importance of ongoing training and awareness strategies to ensure the safety and health of these professionals and contamination in the hospital environment.

## Conclusion

There was a notable improvement in adherence to standard precautions due to the behaviors reported, which include the proper use of PPE, hand hygiene, proper disposal of materials, and measures to prevent contamination of the environment, as evidenced statistically by the increase in the SP adherence score.

The descriptive analysis comparing the percentage of use of PPE in specific respiratory precautions shows that adherence consistently reached 100% for items such as caps, long-sleeved aprons (not impermeable), procedure gloves, and N95 or PFF2 masks.

Also noteworthy is the observation of the results of the Observed Behavior, with emphasis on changes in behavior, such as correct hand hygiene, proper use of the apron during the dressing and undressing process, ensuring that the mask adequately covers the nose, mouth, and chin, proper way of dressing and undressing procedure gloves in the context, concerning adherence before and during dressing and undressing for standard precautions.

Thus, it was possible to verify that the educational intervention carried out by means of a theoretical-practical workshop, using active methodologies, based on the needs of the workers, led to an improvement in adherence to the biosafety measures evidenced both by the behavior reported and by direct observation.

It can be concluded that the use of the educational intervention with biosafety guidelines aimed at hospital cleaning and sanitizing workers was effective and contributed to achieving the highest percentages of knowledge and individual and collective behavior change.

Furthermore, the role of hospital managers is crucial in designing and implementing strategies that support the continuous development of cleaning and hygiene workers. Effective leadership and the commitment of managers are fundamental to ensuring the continuity of education and compliance with biosafety practices, guaranteeing not only the safety of professionals but also the protection of patients from infections in healthcare services.

It is suggested that further studies be carried out focusing on the health of the professionals responsible for cleaning and sanitizing hospitals, including the implementation of educational programs aimed at this group. In addition, it is important to establish connections between the activities carried out by these professionals and patient safety concerning infections in healthcare services.
