# Supplementary material for: Educational intervention on biosafety with hospital hygiene and cleaning workers
Source: Rev Lat Am Enfermagem. 2025 May 19;33:e4518. doi: 10.1590/1518-8345.7449.4518 (PMC12088659; doi:10.1590/1518-8345.7449.4518)
Supplement: Translation

# Translation: Intervenção educativa sobre biossegurança com trabalhadores de higienização e limpeza hospitalar*

**DOI:** 10.1590/1518-8345.7449.4519

**Authors:** Ana Carolina Sobota Vasconcelos, Maria Alice Santos Lobo, Claudia Aparecida Godoy Rocha, Renan Sallazar Ferreira Pereira, Leidiene Ferreira Santos, Mirian Cristina dos Santos Almeida

**Affiliations:** 1 Universidade Federal do Tocantins, Campus Palmas, Palmas, TO, Brasil; 2 Universidade Federal de São João Del-Rei, Campus Centro-Oeste Dona Lindu, Divinópolis, MG, Brasil

**Keywords:** Educação Continuada, Saúde Ocupacional, Zeladoria Hospitalar, Equipamento de Proteção Individual, Exposição Ocupacional, Capacitação em Serviço

## Abstract

avaliar a efetividade de uma intervenção educativa com trabalhadores da limpeza e higienização hospitalar na proteção de agravos por agentes biológicos.

estudo prospectivo, quase experimental, do tipo antes e depois, com grupo único, com a análise do comportamento dos trabalhadores na adesão ao cumprimento às precauções-padrão. A coleta de dados ocorreu com 106 trabalhadores da limpeza e higienização por meio de questionário demográfico e ocupacional e de conhecimento e comportamento referido sobre a prevenção de agravos por agentes biológicos. Realizada a observação do comportamento dos participantes relacionados à biossegurança, pré e pós-intervenção educativa. Análise estatística descritiva e inferencial.

77,4% dos trabalhadores são do sexo feminino, aproximadamente 42% têm até um ano de serviço. Quanto ao grau de escolaridade, 39,6% do Ensino Médio completo e 29,2% Ensino Fundamental incompleto. Mediana do escore de adesão às precauções-padrão: pré-intervenção 14 pontos e pós-intervenção 17 pontos. Dentre a observação antes da paramentação a adesão da higienização das mãos com água e sabão de 41,7% para 75,0% após a intervenção.

a intervenção educativa, fundamentada em oficinas teórico-práticas e metodologias ativas, melhorou a adesão às práticas de biossegurança, evidenciada tanto pelo comportamento relatado quanto pela observação direta. A liderança e o compromisso dos gestores hospitalares são cruciais para a continuidade da educação e as práticas de biossegurança, assegurando a segurança dos profissionais e pacientes. Estudos futuros devem focar na saúde dos trabalhadores de limpeza e higienização hospitalar, incluindo os programas educativos e a relação entre suas atividades e a segurança dos pacientes contra as infecções nos serviços de saúde.

## Destaques

**(1)** Inclusão dos profissionais de higienização e limpeza hospitalar para biossegurança. **(2)** Educação permanente para a promoção da biossegurança entre trabalhadores da limpeza. **(3)** Educação permanente por meio de metodologias ativas. **(4)** Educação permanente promove a adesão aos EPIs em trabalhadores da limpeza hospitalar.

## Introdução

Biossegurança é definida pelas ações destinadas à prevenção, ao controle, à redução ou à eliminação de riscos advindos das atividades que possibilitem o comprometimento da qualidade de vida, da saúde humana e do meio ambiente^(1)^. Os riscos se classificam em físicos, biológicos, químicos, ergonômicos ou de acidente, os quais são meios de contaminação seja em laboratórios de pesquisa ou em unidades de saúde^(2)^.

Os microrganismos patogênicos representam uma preocupação significativa em ambientes fechados, especialmente em serviços como hospitais, devido ao risco de provocar infecções hospitalares^(3)^. Portanto, os profissionais que atuam nesses ambientes estão mais suscetíveis às doenças infecciosas devido à exposição frequente a vírus, bactérias e fungos durante o trabalho^(4)^.

Nessa perspectiva, é notável a importância do uso de Equipamentos de Proteção Individual (EPIs) por funcionários hospitalares, considerando a Norma Regulamentadora (NR) nº 6 do Ministério do Trabalho que define que o EPI é “todo o dispositivo ou produto, de uso individual, utilizado pelo trabalhador, destinado à proteção de riscos à segurança e saúde no trabalho”^(5)^.

Destaca-se que os trabalhadores de limpeza e higienização hospitalar manuseiam diariamente potenciais agentes infectantes que podem se configurar em grande núcleo contaminador, caso não gerenciados corretamente^(6)^. Apesar do reconhecimento global da importância da limpeza em cuidados de saúde, os trabalhadores da limpeza continuam subvalorizados, sendo frequentemente ignorados pelo pessoal clínico em enfermarias movimentadas^(7)^.

Da mesma forma, implementar abordagens de educação em saúde dos agentes de limpeza pode aprimorar seu conhecimento sobre a biossegurança, contribuindo, assim, para um controle mais eficaz de infecções em ambientes de saúde^(8)^.

Para tanto, destaca-se a importância da Educação Permanente em Saúde (EPS) na construção do aprendizado no cotidiano da equipe de trabalho. A EPS abrange as atividades educativas em saúde, gestão e controle social, visando analisar criticamente as necessidades dos usuários e promover mudanças na realidade do trabalho em saúde^(9-9)^. Fortalecer a cultura de segurança dentro das instalações de saúde e fornecer treinamento contínuo são componentes essenciais da EPS, contribuindo para a proteção dos trabalhadores^(11)^.

Diante desse contexto, torna-se evidente a necessidade de estudos que abordem a EPS com trabalhadores de limpeza e higienização hospitalar, com foco em biossegurança. Assim, surgem as questões norteadoras: “Qual a efetividade de uma intervenção educativa na adesão às medidas de biossegurança por trabalhadores de limpeza e higienização hospitalar na proteção contra os agravos causados por agentes biológicos?” e “Atividades de EPS podem contribuir na proteção destes trabalhadores de agravos causados por agentes biológicos?”.

As contribuições deste estudo para a área hospitalar são múltiplas. Primeiramente, ao evidenciar a importância de intervenções educativas na adesão às práticas de biossegurança, o estudo poderá proporcionar um ambiente mais seguro, tanto para os profissionais de saúde quanto para os pacientes. Além disso, a formação contínua e específica dos trabalhadores de limpeza e higienização poderá reduzir a incidência de infecções hospitalares, melhorando a qualidade do atendimento e os resultados clínicos. Por fim, ao promover a conscientização e o treinamento adequado, espera-se que este estudo contribua para a construção de uma cultura organizacional voltada à segurança e à prevenção de riscos, fortalecendo as políticas institucionais de saúde e segurança no trabalho.

Portanto, o objetivo deste estudo foi avaliar a efetividade de uma intervenção educativa com os trabalhadores de limpeza e higienização hospitalar na proteção contra os agravos causados por agentes biológicos.

## Método

### Delineamento do estudo

Trata-se de um estudo prospectivo, quase experimental, do tipo antes e depois, de grupo único, com a análise do comportamento dos trabalhadores na adesão ao cumprimento às precauções-padrão para a proteção de agravos causados por agentes biológicos. A redação deste estudo foi norteada pelo *checklist* do *Strengthening the Reporting of Observational Studies in Epidemiology (Standards for Quality Improvement Reporting Excellence)* (SQUIRE)^(12)^.

### Cenário da pesquisa

O estudo foi desenvolvido em uma unidade de saúde de grande porte, localizada no estado de Tocantins, Brasil. A instituição atende 100% pelo Sistema Único de Saúde (SUS), e é caracterizada como hospital de ensino, geral, público, de nível terciário. Este foi selecionado por tratar-se de um hospital escola e local onde as equipes de trabalhadores da limpeza e higienização atuam em ambientes com alto risco de infecção por material biológico. Os setores que fizeram parte desta pesquisa foram: pronto-socorro adulto, centro cirúrgico e internações.

### População, critérios de seleção e definição da amostra

Todos os trabalhadores da limpeza e higienização hospitalar atuantes nas unidades da instituição foram convidados a participar do estudo. Adotaram-se como critérios de inclusão: ser trabalhador da limpeza e higienização hospitalar vinculado ao hospital pesquisado, não estar com desvio de função ou em cargo de chefia. Foram excluídos os que estavam afastados por qualquer motivo durante o período da coleta de dados e os que não participaram da intervenção educativa.

Para as etapas de observação, utilizou-se como critério de elegibilidade a participação nas demais etapas da pesquisa. Para o cálculo do tamanho amostral considerou-se a população 123 trabalhadores, adotou-se o intervalo de confiança de 99%, erro amostral de 5%, resultando em uma amostra mínima de 104 trabalhadores. Considerou-se as possíveis perdas devido a recusas ou afastamentos, e todos os trabalhadores presentes no ambiente laboral foram convidados a participar do estudo. Na pré-intervenção participaram 115 trabalhadores por meio de respostas aos questionários; na intervenção educativa participaram 106 trabalhadores, assim como na pós-intervenção.

Assim, 106 trabalhadores participaram de todas as etapas, constituindo a amostra final composta por aqueles que participaram tanto da etapa pré-intervenção quanto da intervenção e pós-intervenção.

Para a observação dos pesquisadores sobre a adesão às medidas de biossegurança na atividade laboral pelos trabalhadores da limpeza e higienização hospitalar, tanto na etapa de pré-intervenção, quanto na pós-intervenção, foram observados 12 trabalhadores; essa amostra aleatória simples representou cerca de 10% do total de participantes da primeira etapa da pesquisa.

### Instrumentos utilizados

O instrumento de coleta de dados foi composto por um questionário, constituído por três partes: (1) demográfico e ocupacional; (2) conhecimento e comportamento referido sobre a prevenção de agravos por agentes biológicos; e (3) indagações sobre os tipos de EPI utilizados para as precauções baseadas na transmissão.

O questionário demográfico e ocupacional foi construído pelos autores deste estudo, contendo os seguintes itens: sexo biológico, idade, escolaridade, turno de trabalho e tempo de trabalho, participação prévia em capacitação sobre EPI (pré-intervenção).

O questionário de conhecimento e comportamento referido sobre a prevenção de agravos por agentes biológicos (pré e pós-intervenção) foi adaptado da escala da versão brasileira da *Compliance With Standard Precautions Scale* (CSPS-PB)^(13)^, validada por meio de processos metodológicos como: tradução, consenso entre juízes, retrotradução, validação semântica e pré-teste.

A escala CSPS representa as alternativas para medir a adesão dos profissionais de Enfermagem na prática de controle da infecção. Devido à falta de instrumentos específicos para os trabalhadores da limpeza e higienização hospitalar e buscando a adequação a sua atividade laboral, o questionário adaptado a partir do CSPS foi mantido com 20 itens. Desses, os itens 3, 6, 7, 12, 13, 14, 15, 16 e 17 permaneceram inalterados em relação ao original. Os itens 1, 8, 10 e 19 foram modificados, enquanto os itens 2, 4, 5, 9, 11, 18 e 20 foram removidos. Além disso, foram adicionados sete novos itens relacionados ao uso de máscaras, paramentação e desparamentação dos EPIs. Previamente, o pré-teste foi realizado com alguns trabalhadores da limpeza e higienização hospitalar da instituição onde ocorreu o estudo.

Ao seguir os mesmos pressupostos da CSPS-PB, o questionário ficou com quatro opções de respostas que indicaram a frequência do cumprimento às precauções-padrão (PP), compreendendo “sempre”, “muitas vezes”, “raramente” ou “nunca”. Considerou-se, ainda, que os profissionais deveriam aderir integralmente às PP, sendo esperado que a opção “sempre” totalizasse a maioria das respostas^(13)^.

O escore de cumprimento às PP varia de zero a 20, e quanto mais próximo de 20, melhor a adesão dos profissionais. Para a opção de resposta “sempre” foi atribuído o escore de “um” e para as opções “muitas vezes”, “raramente” ou “nunca” o escore atribuído foi “zero”.

Para registrar a observação do comportamento dos participantes (pré e pós-intervenção) utilizou-se um formulário denominado *Checklist* de avaliação da adesão às medidas de prevenção de agravos por agente biológicos/utilização de EPIs. Este instrumento é uma versão adaptada do *Checklist* dos EPIs para a paramentação e desparamentação^(14-14)^.

### Coleta de dados

A coleta de dados foi feita no período de dezembro de 2022 a abril de 2023 por pesquisadores previamente capacitados, sendo realizada em quatro etapas, distribuídas durante a pré-intervenção, intervenção e pós-intervenção.

Na primeira etapa realizou-se uma reunião com os trabalhadores de cada turno de trabalho para a apresentação do estudo, convite e aplicação do questionário sobre o perfil demográfico e ocupacional, e sobre o conhecimento e o comportamento referido em relação à prevenção de agravos por agentes biológicos.

Os instrumentos respondidos foram colocados pelo próprio participante em um envelope sem identificação, lacrado e entregue aos pesquisadores ao término do preenchimento. Durante a coleta de dados, os pesquisadores ficaram disponíveis para as eventuais dúvidas no preenchimento do questionário.

A segunda etapa compreendeu a fase de observação do comportamento dos participantes (pré e pós-intervenção) para a avaliação da adesão às medidas de prevenção de agravos por agentes biológicos/utilização de EPIs. Ocorreu em seis dias de campo, totalizando 72 horas, sendo possível realizá-la em todos os turnos.

Foram observadas as diferentes dinâmicas e rotinas do serviço, em horários e períodos alternados, considerando as possibilidades apresentadas pelo campo. Essa aproximação foi fundamental para a clareza, a identificação e a compreensão da organização e da dinâmica do processo de paramentação e desparamentação no cenário estudado.

Os observadores evitaram estabelecer contato direto com os participantes para não influenciar seu comportamento. Na observação dos participantes e registro dos comportamentos, cada trabalhador foi observado por dois pesquisadores durante momentos alternados^(16)^.

Na terceira etapa da coleta de dados, foi implementada a intervenção educativa. Para tanto, elaborou-se um plano de ensino de acordo com a necessidade de educação permanente identificada por meio dos questionários aplicados e a observação dos participantes.

Esta etapa foi realizada no formato de oficina teórico-prática, com a utilização de metodologias ativas, contendo as seguintes temáticas: técnica de higienização das mãos; riscos biológicos e vias de contaminação; sequência indicada para paramentação e desparamentação; tipos de precauções; e EPI conforme o tipo de precaução.

Como parte das metodologias ativas utilizou-se a prática de higienização das mãos na “Caixa da verdade”; Teatro “Simulação do risco de contaminação”, onde elucidou-se sobre a transmissibilidade de agentes biológicos; Oficina de paramentação e desparamentação e “Jogo dos Erros”, por meio de imagens e simulações de situações de trabalho em que o trabalhador apontava os erros relativos à utilização de EPIs.

Na quarta etapa, após um mês da realização da intervenção educativa, foi efetuado o pós-teste, aplicando-se o questionário de conhecimento e comportamento referido sobre a prevenção de agravos por agente biológicos e observação dos participantes pós-intervenção.

### Intervenção educativa

A intervenção educativa com os trabalhadores de limpeza e higienização hospitalar foi desenvolvida após a identificação do conhecimento prévio e observação das práticas relacionadas à biossegurança.

Optou-se pelo formato de oficinas teórico-práticas, trabalhando momentos dinâmicos para a assimilação dos conteúdos explanados. As atividades realizadas nessas dinâmicas foram:

*Simulação da higienização das mãos na “Caixa da verdade”* - Consiste em uma caixa organizadora com uma abertura lateral para a inserção das mãos e uma abertura na tampa para visualização. Na lateral interna direita, incorporou-se uma luz negra de cinco watts. Para realçar a iluminação proveniente da luz negra, o interior da caixa foi revestido com cor preta.

Como método de higienização, adotou-se uma solução de álcool em gel 70%, adicionada a uma tintura transparente que se torna fluorescente na presença da luz negra. Essa solução foi disponibilizada aos profissionais para a higienização das mãos.

Após a aplicação, os profissionais foram conduzidos até a “Caixa da verdade” para verificar a cobertura das mãos pela solução alcoólica fluorescente, identificando a presença de áreas não higienizadas adequadamente.

*Peça teatral* - Durante a demonstração realizada pelo membro da equipe de pesquisa, foi apresentada a simulação da transmissão de agentes biológicos. A cena retratou o profissional recolhendo o lixo contaminado, com o saco de lixo previamente sujo com tinta guache para representar os referidos agentes biológicos.

Na encenação, após coletar o lixo, o profissional abre uma porta e toca em sua máscara, coçando o nariz com as luvas que manipularam o saco de lixo contaminado. Posteriormente, ao remover as luvas, o profissional omite a higienização das mãos, cumprimenta um colega de trabalho com um aperto de mãos e continua suas atividades laborais.

*Segurança do trabalho -* Esta dinâmica envolveu o uso de dois balões, ambos inflados com ar, sendo que apenas um deles foi envolto por fita adesiva transparente. Foi explicado que o balão sem fita representava o profissional que não utiliza os EPIs ou o faz de maneira inadequada, enquanto o balão com fita representava os profissionais que utilizam os EPIs corretamente.

Uma agulha simbolizou o acidente de trabalho e os riscos de contaminação. Ao introduzir a agulha no balão sem fita, este se rompeu facilmente, enquanto ao inseri-la no balão com a fita, o balão permaneceu intacto.

*Jogo dos erros* - Foram exibidas imagens ou simulações de situações de trabalho que evidenciavam os equívocos no uso de EPIs, apresentando os riscos de contaminação, assim como inadequações nas práticas de paramentação e desparamentação. Os trabalhadores identificaram os erros nas cenas e apontaram as ações corretas apropriadas para cada situação.

*O que está faltando?* – Foram apresentados atores (pesquisadores) paramentados com EPIs, conforme as indicações de todas as precauções. Os trabalhadores, divididos em grupos, observaram e identificaram, verbalmente, quais EPIs estavam faltando.

Assim, a oficina pedagógica foi concebida com fundamentos na aprendizagem significativa, abordagem multimodal e a aplicação de metodologias ativas. Incluíram-se discussões sobre os temas apresentados, bem como os momentos dedicados às atividades práticas, visando proporcionar uma compreensão mais aprofundada para os trabalhadores na área de limpeza e higienização hospitalar.

### Análise dos dados

A análise descritiva foi realizada por frequências absolutas e relativas para as variáveis qualitativas, e por médias e desvios-padrão para as quantitativas. O teste de McNemar foi utilizado para comparar aos itens que compõem a PP nos momentos pré e pós-intervenção, e o escore obtido pela pontuação da adesão foi comparado pelo teste de Wilcoxon, por não ter sido observada a aderência dos dados à distribuição normal, que por sua vez foi avaliada pelo teste de Shapiro-Wilk. O nível de significância foi de 5%. O programa utilizado foi o *Stata* (*StataCorp*, LC) versão 15.0.

Os registros da observação realizados pelos dois pesquisadores tiveram como grau de concordância obtido o coeficiente *kappa* 0,94, que evidenciou uma alta concordância.

### Aspectos éticos

Este estudo foi realizado em consonância com a Resolução nº 466/12 do Conselho Nacional de Saúde (CNS)^(17)^. O projeto obteve a autorização institucional para coleta de dados e foi apreciado e aprovado pelo Comitê de Ética em Pesquisa (CEP) da Universidade Federal do Tocantins (UFT) (CAAE-63028922.2.0000.5519/Parecer 5.694.479).

## Resultados

A idade média dos participantes do estudo foi de 40,9 anos (dp 11,9), sendo a idade mínima 21 anos e a máxima 67 anos. A Tabela 1 apresenta o perfil demográfico e ocupacional dos 106 trabalhadores da limpeza e higienização hospitalar.

**Tabela 1 -:** Distribuição numérica e percentual do perfil demográfico e ocupacional dos trabalhadores da limpeza e higienização hospitalar (n = 106). Tocantins, TO, Brasil, 2022-2023

Variáveis	n (%)
**Sexo biológico**	
Feminino	82 (77,4)
Masculino	24 (22,6)
**Escolaridade**	
Fundamental completo	10 (9,4)
Fundamental incompleto	31 (29,2)
Médio completo	42 (39,6)
Médio incompleto	21 (19,8)
Superior completo	1 (0,9)
Superior incompleto	1 (0,9)
**Turno de trabalho**	
Diurno	63 (59,4)
Noturno	43 (40,6)
**Tempo de trabalho (anos)**	
Até 1	40 (37,8)
1 a < 2	27 (25,4)
2 a < 3	7 (6,6)
3 ou mais	32 (30,2)
Participação anterior em capacitações sobre EPI*	
Sim	95 (89,6)
Não	11 (10,4)
**EPI Teórica/Prática**	
Teórica	75 (70,7)
Prática	26 (24,6)
Ambas	5 (4,7)
Quantas vezes participou de capacitações sobre EPI*	
01-2	63 (59,4)
03-4	(19,9)
5+	22 (20,7)

*Equipamento de Proteção Individual

Dentro do espectro do sexo biológico, evidencia-se uma marcante predominância feminina, ou seja, 77,4%. Em relação à formação educacional, cerca de 40% dos profissionais alcançaram a culminância do Ensino Médio, e cerca de um terço não obteve a conclusão do Ensino Fundamental. Uma mera fração, 1,8%, ascendeu ao Ensino Superior. Notavelmente, 59,4% dos entrevistados operavam em turno diurno.

Ao se considerar o tempo de trabalho, aproximadamente 42% têm até um ano de serviço, com um quarto situando-se entre um e dois anos de atividade. O restante distribui-se entre dois e três anos, ou ultrapassa o marco de três anos.

No cerne da capacitação profissional, um destaque é a formação em EPIs. Em relação a esta variável 89,6% já foi imerso em treinamentos anteriores. Entretanto, identifica-se que 70,7% são de natureza teórica, embora cerca de um quarto tenham tido uma vertente prática ou teórico-prática.

A Tabela 2 ilustra o percentual de adesão pré e pós-intervenção educativa para cada item do questionário de adesão às precauções-padrão.

**Tabela 2 -:** Adesão ao cumprimento às precauções-padrão nos momentos pré e pós-intervenção por trabalhadores da limpeza e higienização hospitalar (n = 106). Tocantins, TO, Brasil, 2022-2023

**Questões**	**Pré-intervenção**	**Pós-intervenção**	p*
1. Eu lavo minhas mãos quando pego em maçanetas e equipamentos contaminados.	72 (67,9%)	106 (100,0%)	<0,001
2. Eu uso produto a base de álcool para higienizar as mãos como uma alternativa, se não estiverem visivelmente sujas.	69 (65,1%)	96 (90,6%)	<0,001
3. A caixa de materiais perfurocortantes é descartada somente quando está cheia.	96 (90,6%)	106 (100,0%)	0,002
4. Eu retiro os EPIs ^†^ em um local designado.	59 (55,7%)	86 (81,1%)	<0,001
5. Eu tomo banho e troco roupa privativa em caso de grandes respingos, mesmo que eu tenha usado EPI ^†^ .	63 (59,4%)	83 (78,3%)	0,002
6. Eu uso luvas de borracha de cano longo quando estou exposto a fluidos corporais, sangue ou derivados e qualquer excreção de pacientes.	52 (49,1%)	0 (0%)	<0,001
7. Eu higienizo as minhas mãos imediatamente após remover as luvas.	92 (86,8%)	106 (100,0%)	<0,001
8. Eu uso máscara cirúrgica em combinação com óculos de proteção e avental sempre que houver a possibilidade de respingos ou derramamentos.	40 (37,7%)	75 (70,8%)	<0,001
9. Minha boca e meu nariz ficam cobertos quando uso máscara.	95 (89,6%)	106 (100,0%)	0,001
10. Eu reutilizo uma máscara cirúrgica ou EPI ^†^ descartável.	71 (67,0%)	106 (100,0%)	<0,001
11. Eu uso avental/capote quando estou exposto a sangue, fluidos corporais ou a qualquer excreção de pacientes.	68 (64,2%)	106 (100,0%)	<0,001
12. Eu descarto material contaminado com sangue, fluidos corporais, secreções e excreções de pacientes em sacos plásticos brancos, independente do estado infeccioso do paciente.	98 (92,5%)	106 (100,0%)	0,008
13. Eu uso luvas de procedimento para descontaminar equipamentos que apresentam sujeira visível.	94 (88,7%)	106 (100,0%)	<0,001
14. Eu evito tocar olhos, nariz e boca com as mãos não lavadas/higienizadas.	93 (87,7%)	106 (100,0%)	<0,001
15. Eu evito tocar superfícies (ex. mobiliário, maçanetas e equipamentos para a saúde) com luvas ou outros EPIs ^†^ contaminados ou com as mãos contaminadas.	78 (73,6%)	106 (100,0%)	<0,001
16. Eu evito tocar na parte da frente da máscara e quando isso ocorre, imediatamente higienizo as mãos.	67 (63,2%)	106 (100,0%)	<0,001
17. Eu removo a máscara usando a técnica apropriada (ou seja, não toco na frente da máscara, mas removo pelas tiras).	74 (69,8%)	106 (100,0%)	<0,001
18 Eu realizo a higienização das mãos após a remoção da máscara.	86 (81,1%)	106 (100,0%)	<0,001
19. Após a utilização eu descarto os EPIs ^†^ descartáveis em lixo para resíduo infectante (saco branco).	86 (81,1%)	106 (100,0%)	<0,001
20. Após a utilização, eu retiro o avental procurando segurar na parte interna do mesmo, evitando me contaminar.	75 (70,8%)	106 (100,0%)	<0,001

*McNemar p = Valor de p para comparação entre pré e pós-intervenção educativa; ^†^Equipamento de Proteção Individual

Inicialmente, destaca-se que na comparação do comportamento referido antes e após a intervenção educativa, todos os itens apresentaram positivamente uma diferença estatisticamente significativa, com valor de p< 0,001. O resultado contrasta com o item 7, onde o “uso luvas de borracha quando estou exposto a fluidos corporais, sangue ou derivados e qualquer excreção de pacientes” passou a ser zero após a intervenção, devido à ausência do EPI no serviço (substituído por luva de látex).

A mediana do escore global de adesão às PP, comparada entre pré e pós-intervenção, foi maior na pós-intervenção com 17 pontos (Figura 1).

A Tabela 3 apresenta a adesão aos EPIs específicos para as precauções respiratórias antes e depois da intervenção.

**Figura 1 -:** Efeitos da intervenção educativa sobre a pontuação da adesão às precauções padrão dos trabalhadores da limpeza e higienização hospitalar (n = 106). Tocantins, TO, Brasil, 2023

**Tabela 3 -:** Adesão aos EPIs* para a precaução específica respiratória nos momentos pré e pós-intervenção pelos trabalhadores da limpeza e higienização hospitalar (n = 106). Tocantins, TO, Brasil, 2023

Variáveis	Pré				Pós			
	**Sempre**	**Às vezes**	**Raramente**	**Nunca**	**Sempre**	**Às vezes**	**Raramente**	**Nunca**
***Transmissão respiratória- aerossóis***								
Gorro	99 (93,4%)	3 (2,8%)		4 (3,8%)	106(100,0%)			
Máscara cirúrgica	24 (22,6%)	1 (0,9%)	4 (3,8%)	77 (72,7%)				106 (100,0%)
Avental de mangas longas (não impermeável)	80 (75,5%)	10 (9,4%)	7 (6,6%)	9 (8,5%)	106(100,0%)			
Luvas de procedimento	93 (87,8%)	7 (6,6%)	1 (0,9%)	5 (4,7%)	106(100,0%)			
Máscara N95 ou PFF2 ^†^	88 (83,0%)	14 (13,2%)		4 (3,8%)	106(100,0%)			
Óculos de proteção ou protetor facial	31 (29,2%)	13 (12,3%)	9 (8,5%)	53 (50,0%)				106 (100,0%)
Avental mangas longas impermeável	33 (31,1%)	11 (10,4%)	9 (8,5%)	53 (50,0%)				106 (100,0%)
Teste de vedação da N95, PFF2 ^†^ ou similar	65 (61,3%)	12 (11,3%)	8 (7,6%)	21 (19,8%)	84 (79,3%)	15 (14,1%)	2 (1,9%)	5 (4,7%)
***Transmissão respiratória-gotículas***								
Máscara cirúrgica	80 (75,5%)	6 (5,7%)	2 (1,8%)	18 (17,0%)	106 (100,0%)			
Avental de mangas longas (não impermeável)	80 (75,5%)	15 (14,2%)	1 (0,9%)	10 (9,4%)	106 (100,0%)			
Luvas de procedimento	93 (87,7%)	9 (8,5%)	1 (0,9%)	3 (2,9%)	106 (100,0%)			
Óculos de proteção ou protetor facial	32 (30,2%)	13 (12,3%)	8 (7,5%)	53 (50,0%)				106 (100,0%)
Avental mangas longas impermeável	36 (34,0%)	6 (5,7%)	8 (7,5%)	56 (52,8%)				106 (100,0%)
Avental pano ou lona padrão	19 (17,9%)	9 (8,5%)	8 (7,6%)	70 (66,0%)				106 (100,0%)

*Equipamento de Proteção Individual; ^†^Peça Facial Filtrante Classe 2

Pela análise descritiva simples, observa-se um aumento no percentual de adesão de diversos EPIs, tanto para as precauções com aerossóis, quanto para gotículas, com destaque para itens como gorro, avental de mangas longas (não impermeável), luvas de procedimento, e máscara N95 ou Peça Facial Filtrante Classe 2 (PFF2), a adesão passou a ser de 100% “sempre”.

No âmbito da transmissão respiratória por aerossóis, a adesão ao uso contínuo de gorros, aventais de mangas longas (não impermeáveis), luvas de procedimento, máscaras N95 ou PFF2 após a intervenção foi de 100%. Antes da intervenção, a utilização de máscaras cirúrgicas era baixa, com apenas 22,6% dos profissionais usando-as sempre, e 72,6% nunca as utilizando. No entanto, no período pós-intervenção, o uso desse EPI caiu para zero, sendo esse o resultado esperado, visto que o protocolo é a não utilização de máscara cirúrgicas em precauções de transmissão respiratória por aerossóis.

Nota-se que, em relação ao teste de vedação da N95, PFF2 ou similares, houve uma elevação na adesão de 61,3% no período pré-intervenção para 79,3% no pós-intervenção.

Em relação à transmissão respiratória por gotículas, observa-se que 75,5% dos profissionais sempre usavam máscaras cirúrgicas e aventais de mangas longas (não impermeáveis) no momento da pré-intervenção.

Na pós-intervenção, essa taxa alcançou 100%. As luvas de procedimento eram frequentemente usadas por 87,7% dos entrevistados antes da intervenção, valor que posteriormente também ascendeu a 100%. Por outro lado, antes da intervenção 30,2% dos profissionais sempre utilizavam óculos de proteção ou protetor facial e 34,0% faziam uso constante de avental impermeável de mangas longas.

Após a intervenção, ambas as práticas foram completamente descontinuadas. O avental padrão de pano ou lona tinha uma adesão de apenas 17,9% no pré-intervenção, e tal como os outros EPIs mencionados, seu uso também cessou no pós-intervenção.

A Tabela 4 apresenta os itens observados pelos pesquisadores nos atos anteriores à paramentação, durante a mesma e na desparamentação dos trabalhadores da limpeza e higienização, quando de forma aleatória foram observados o comportamento de 12 profissionais antes e após a intervenção, em relação às medidas de biossegurança.

**Tabela 4 -:** Procedimentos observados antes e durante a paramentação e desparamentação para precauções-padrão nos momentos pré e pós-intervenção educativa pelos trabalhadores da limpeza e higienização hospitalar (n = 12). Tocantins, TO, 2022-2023

Antes da paramentação	Pré-intervenção	Pós-intervenção
Higieniza as mãos com água e sabão	5 (41,7%)	9 (75,0%)
Separa e valida a integridade dos EPI*	11 (91,7%)	12 (100,0%)
***Avental:*** prende os laços do pescoço e da cintura	7 (58,3%)	12 (100,0%)
**Durante a paramentação**	Pré-intervenção	Pós-intervenção
***Máscara cirúrgica***		
Usa máscara cirúrgica	11 (91,7%)	12 (100,0%)
Posiciona o clipe nasal sobre o dorso do nariz	11 (91,7%)	12 (100,0%)
Posiciona os elásticos atrás das orelhas ou amarra as fitas	11 (91,7%)	12 (100,0%)
Assegura que a máscara cobre o nariz, a boca e o queixo	7 (58,3%)	10 (83,3%)
Óculos		
Não usa	12 (100,0%)	12 (100,0%)
***Protetor facial***		
Não usa	12 (100,0%)	12 (100,0%)
***Calça as luvas de procedimento*** cobrindo os punhos das mangas do avental	12 (100,0%)	12 (100,0%)
Coloca o ***Gorro*** cobrindo os cabelos e as orelhas	11 (91,7%)	12 (100,0%)
***Bota de borracha de cano longo***		
Não usa	12 (100,0%)	12 (100,0%)
**Durante a desparamentação**		
***Luvas de procedimento***		
Segura no punho da luva da mão não dominante e retira a primeira luva	8 (66,7%)	12 (100,0%)
Posiciona o dedo indicador sob o punho da luva e retira a segunda luva	1 (8,3%)	12 (100,0%)
Não	11 (91,7%)	0 (0,0%)
Descarta as luvas no lixo infectante	12 (100,0%)	12 (100,0%)
***Higieniza as mãos*** com preparação alcoólica a 70%	6 (50,0%)	12 (100,0%)
***Avental***		
Remove primeiro o laço do pescoço e depois da cintura	5 (41,7%)	11 (91,7%)
Posiciona o dedo indicador na parte interna do punho e puxa o avental cobrindo a mão não dominante	2 (16,7%)	8 (66,7%)
Com a mão protegida pela manga do avental, segura a outra manga, enquanto desliza e retira o braço e a mão dominante do avental	1 (8,3%)	10 (83,3%)
Segura na parte interna do avental (próximo ao ombro) e retira a manga do braço não dominante	1 (8,3%)	10 (83,3%)
Continua virando o avental de dentro para fora, dobrando e formando um pacote	1 (8,3%)	10 (83,3%)
Descarta o avental no lixo infectante	9 (75,0%)	12 (100,0%)
***Higieniza as mãos*** com preparação alcoólica a 70%	6 (50,0%)	12 (100,0%)
*Óculos ou protetor*		
Não usa	12 (100,0%)	12 (100,0%)
***Gorro-estava usando***	11 (91,7%)	12 (100,0%)
Posiciona os dedos indicadores dentro do gorro ao lado das orelhas e desliza o gorro para cima, para trás e para o lado	8 (66,7%)	12 (100,0%)
Descarta o gorro no lixo infectante	11 (91,7%)	12 (100,0%)
***Higieniza as mãos*** com preparação alcoólica a 70%	1 ( 8,3%)	10 (83,3%)
***Máscara cirúrgica***		
Estava usando	9 (75,0%)	12 (100,0%)
Segura nos elásticos atrás das orelhas, ou desfaz os laços das fitas na região posterior da cabeça	9 (75,0%)	12 (100,0%)
Puxa a máscara para frente, segurando nos elásticos ou nas fitas, sem tocar em sua superfície	8 (66,6%)	12 (100,0%)
Descarta a máscara no lixo infectante	9 (75,0%)	12 (100,0%)
Higieniza as mãos com preparação alcoólica a 70%	0 (0,0%)	9 (75,0%)
***Bota de borracha impermeável de cano longo***		
Não estava usando	12 (100,0%)	12 (100,0%)

*Equipamento de Proteção Individual

Observa-se que todos os itens avaliados “antes da paramentação” tiveram aumento de adesão pós-intervenção educativa.

Durante a paramentação, todos os procedimentos associados à máscara cirúrgica, incluindo seu uso, posicionamento e ajuste, exibiram uma adesão de 100% após a intervenção, com exceção do procedimento de garantir que a máscara cobrisse o nariz, boca e queixo, que aumentou de 58,3% para 83,3%. Em contraste, o uso do gorro, que cobre cabelos e orelhas, alcançou 100% de adesão após a intervenção.

Quanto aos procedimentos observados durante a desparamentação, houve aumento na adesão em muitas práticas. No caso da retirada das luvas, a técnica de segurar no punho da luva da mão não dominante para retirar a primeira luva e a de posicionar o dedo indicador sob o punho da luva para retirar a segunda alcançaram 100% de adesão após a intervenção.

A higienização das mãos com preparação alcoólica a 70% depois da desparamentação do avental e da máscara cirúrgica passou de 50,0% e 0,0% para 100,0% e 75,0%, respectivamente. O procedimento de remover o avental, virando-o de dentro para fora e dobrando-o, aumentou de 8,3% para 83,3%.

## Discussão

Este estudo focalizou-se na análise do perfil demográfico e ocupacional dos trabalhadores da limpeza e higienização hospitalar de um hospital no estado de Tocantins, Brasil, em 2022/2023, bem como na efetividade da intervenção de educação permanente sobre a biossegurança.

Em consonância com os estudos anteriores, identificou-se uma predominância feminina na limpeza e higienização hospitalar^(18)^.

Em relação à formação educacional, uma análise minuciosa revelou nuances críticas. A limitada presença de profissionais com educação superior coadunou-se com a literatura, que sugere que essas funções são comumente ocupadas por indivíduos com menos anos de formação acadêmica^(19)^.

A distribuição equitativa entre os turnos de trabalho diurno e noturno pode afetar tanto o bem-estar dos profissionais quanto a eficácia operacional das instituições. Os hospitais funcionam 24 horas por dia, exigindo que os profissionais de limpeza estejam preparados para atuar em turnos que cobrem todo o período, tanto diurno quanto noturno. A manutenção da higiene hospitalar é essencial ao longo do dia, mas o trabalho noturno pode apresentar desafios adicionais para a saúde^(20)^. Os turnos de trabalho para pessoal idoso ou inexperiente devem ser organizados em períodos curtos durante a tarde, visando minimizar o risco de acidentes de trabalho. Além disso, o pessoal mais idoso deve ser alocado em unidades de baixo risco para prevenir acidentes^(21)^.

Os dados destacaram uma alta participação em capacitações anteriores sobre EPIs, com a maior parte delas de natureza teórica. Tal dicotomia no treinamento, concomitante com a frequência de atualizações de EPI, evoca reflexões sobre a efetividade e a qualidade do treinamento oferecido. A carência de treinamento adequado também foi percebida em outros departamentos, onde os participantes declararam não dispor de todas as informações necessárias para desempenhar suas tarefas^(22)^.

Ao reforçar a importância de implementar treinamentos eficazes nas unidades de saúde a partir das necessidades dos trabalhadores, essa abordagem proporciona mais chances de aprendizado, resultando em uma adesão mais ampla dos profissionais às medidas de proteção individual, ao mesmo tempo reduzindo a vulnerabilidade aos riscos^(23)^. Por outro lado, o conhecimento, por si só, não indica necessariamente uma mudança de comportamento, visto que este pode estar relacionado aos fatores intrínsecos e extrínsecos.

Nesse sentido, o letramento em saúde está ligado à aquisição de conhecimento e à transformação de comportamentos individuais. Ele pode ser conceituado como um conjunto de competências fundamentais e recursos contextuais de que as pessoas necessitam para adquirir, compreender, avaliar, comunicar e aplicar as informações e os serviços de maneiras diversas, em vários cenários ao longo de suas vidas, com o propósito de aprimorar sua saúde e o bem-estar^(24)^.

A educação em saúde tem o potencial de promover mudanças nos comportamentos humanos. Ela pode incentivar a reflexão sobre a organização do trabalho de limpeza, identificar os fatores que causam adoecimento e discutir as estratégias para reduzi-los. Isso ajuda a ressignificar as práticas de autocuidado e melhora a capacidade de enfrentar os problemas que afetam a saúde física e mental dos trabalhadores do Serviço Hospitalar de Limpeza^(25)^.

Dessa forma, as competências que vão além do mero conhecimento e treinamento desempenham um papel significativo no desenvolvimento de habilidades práticas, como a paramentação, desparamentação e higienização das mãos, assim como na formação de atitudes positivas. Isso ressalta o impacto benéfico da intervenção realizada por meio de oficinas teórico-práticas que empregam metodologias ativas.

Após a intervenção educativa, observou-se um aumento expressivo na adesão às precauções-padrão. Estes resultados estão em sintonia com a literatura existente, que identifica a efetividade de várias estratégias, incluindo o treinamento, *feedback* e sistemas facilitadores^(26-26)^.

Notou-se um aumento significativo na utilização de álcool como agente higienizador quando as mãos não apresentavam sujidade visível, evidenciado uma lacuna no conhecimento combinada com a falta de prática diária. A higienização das mãos, seja com água e sabonete líquido ou com preparações alcoólicas, em conformidade com os cinco momentos estabelecidos para essa prática nos serviços de saúde, deve ser uma rotina já internalizada por todos os profissionais de saúde. É imperativo reiterar sua importância como uma medida fundamental para a prevenção e o controle de infecções, tanto no presente quanto no futuro^(28)^. Portanto, é crucial que as unidades de saúde ofereçam treinamentos contínuos, visando à conformidade com os padrões de higiene das mãos estabelecidos pelos órgãos competentes^(29-29)^.

A conexão entre a implementação de práticas de biossegurança, a preservação da saúde dos trabalhadores e a segurança dos pacientes é vital. Isso enfatiza que a biossegurança é um procedimento funcional e operacional de extrema relevância nos diversos serviços de saúde, devendo ser reconhecida como um instrumento de proteção, tanto para o paciente quanto para os profissionais envolvidos na prestação de cuidados de saúde^(31)^.

Houve melhorias notáveis nos seguintes aspectos: identificação do local correto para retirar os EPIs, uso adequado dos EPIs ao lidar com fluidos corporais, compreensão das combinações ideais de EPIs para garantir a segurança, tanto do profissional quanto do paciente, consideração sobre a reutilização dos EPIs, utilização dos EPIs para a descontaminação de equipamentos e superfícies e adoção correta de medidas para evitar a contaminação durante o processo de desparamentação. Esta melhoria está alinhada aos estudos anteriores que enfatizaram a proteção proporcionada por tais equipamentos em ambientes hospitalares^(32)^.

Conforme as diretrizes da Agência Nacional de Vigilância Sanitária do Brasil (ANVISA), é recomendado que os profissionais de higiene e limpeza em serviços hospitalares utilizem óculos ou protetor facial (especialmente quando há risco de respingos de materiais orgânicos ou químicos), avental (se houver risco de contato com fluidos ou secreções do paciente que possam ultrapassar a barreira do avental de contato, o profissional deve usar avental impermeável), luvas de borracha de cano longo e botas impermeáveis de cano longo^(28)^.

Quanto ao descarte de EPIs e materiais hospitalares, todos os profissionais demonstraram plena adesão aos itens 3, 12 e 19 no questionário de conhecimento autorreferido. O resultado corrobora outros estudos, em que o descarte impróprio de materiais foi identificado como uma das principais causas de acidentes com material biológico, ocorrendo com maior frequência quando os profissionais não utilizavam EPIs, representando 51% dos casos^(33)^.

A adesão a algumas práticas de EPI antes da intervenção era modesta. Esta baixa adesão pré-intervenção sugeriu a existência de lacunas na sensibilização ou compreensão da importância dos EPIs. Na desparamentação, houve um aumento considerável na adesão aos procedimentos corretos pós-intervenção, alinhando-se com as diretrizes que sublinham a importância deste processo para evitar as contaminações^(34)^.

O estudo se justifica pela escassez de trabalhos científicos focados na população dos trabalhadores de limpeza e higienização hospitalar. Entre as limitações encontradas, destaca-se a falta de questionários validados que abordem as medidas de biossegurança específicas para esses trabalhadores. O questionário de precaução-padrão não menciona os EPIs apropriados para essa categoria, como luvas de borracha e botas de borracha impermeáveis de cano longo. Portanto, sugere-se a realização de estudos para validar os questionários de biossegurança voltados para essa população.

Outra limitação do estudo é a aplicação dos resultados obtidos. A observação dos participantes pode interferir no comportamento deles em relação à adesão às medidas de biossegurança, já que podem perceber que estão sendo observados naquele momento, o que pode alterar suas ações.

As implicações para o profissional são significativas, visto que o estudo destaca a necessidade de desenvolvimento e validação de ferramentas específicas para avaliar a adesão às práticas de biossegurança entre os trabalhadores de limpeza e higienização hospitalar. Além disso, reforça a importância de estratégias de formação e conscientização contínuas para garantir a segurança e a saúde desses profissionais e a contaminação no ambiente hospitalar.

## Conclusão

Houve uma notável melhoria na adesão às PP pelos comportamentos relatados, que incluem o uso adequado de EPI, higienização das mãos, descarte apropriado de materiais e medidas de prevenção da contaminação do ambiente, o que foi evidenciado estatisticamente pelo aumento no escore de adesão às PP.

Pela análise descritiva ao se comparar o percentual de utilização de EPIs em precauções específicas respiratórias, ressalva-se que a adesão atingiu consistentemente 100% para os itens como gorro, avental de mangas longas (não impermeável), luvas de procedimento e máscara N95 ou PFF2.

Também merece destaque a observação dos resultados do comportamento observado, com ênfase nas mudanças de comportamento, como a correta higienização das mãos, o uso apropriado do avental durante o processo de paramentação e desparamentação, a garantia de que a máscara cubra adequadamente o nariz, a boca e o queixo, a forma adequada de paramentação e desparamentação das luvas de procedimento no contexto, em relação à adesão antes e durante a paramentação e desparamentação para as precauções-padrão.

Assim, foi possível verificar que a intervenção educativa realizada por meio de oficina teórico-prática, com a utilização de metodologias ativas, a partir das necessidades dos trabalhadores, proporcionou uma melhora na adesão às medidas de biossegurança evidenciadas, tanto pelo comportamento referido quanto pela observação direta.

Conclui-se que o uso da intervenção educativa com as orientações de biossegurança voltadas aos trabalhadores da limpeza e higienização hospitalar foi efetiva e contribuiu para alcançar maiores percentuais de conhecimento e mudança de comportamento individual e coletivo.

Ademais, o papel dos gestores hospitalares é crucial na elaboração e implementação de estratégias que apoiem o desenvolvimento contínuo dos trabalhadores de limpeza e higiene. A liderança efetiva e o comprometimento dos gestores são fundamentais para assegurar a continuidade da educação e o cumprimento das práticas de biossegurança, garantindo não apenas a segurança dos profissionais, mas também a proteção dos pacientes contra as infecções nos serviços de saúde.

Sugere-se a realização de estudos subsequentes que enfoquem a saúde dos profissionais responsáveis pela limpeza e higienização hospitalar, incluindo a implementação de programas educativos direcionados a esse grupo. Além disso, é relevante estabelecer conexões entre as atividades desempenhadas por esses profissionais e a segurança dos pacientes em relação às infecções nos serviços de saúde.

# Translation: Intervención educativa sobre bioseguridad con los trabajadores de higienización y limpieza hospitalaria*

**DOI:** 10.1590/1518-8345.7449.4517

**Authors:** Ana Carolina Sobota Vasconcelos, Maria Alice Santos Lobo, Claudia Aparecida Godoy Rocha, Renan Sallazar Ferreira Pereira, Leidiene Ferreira Santos, Mirian Cristina dos Santos Almeida

**Affiliations:** 1 Universidade Federal do Tocantins, Campus Palmas, Palmas, TO, Brasil; 2 Universidade Federal de São João Del-Rei, Campus Centro-Oeste Dona Lindu, Divinópolis, MG, Brasil

**Keywords:** Educación Continua, Salud Laboral, Servicio de Limpieza en Hospital, Equipo de Protección Personal, Exposición Profesional, Capacitación em Servicio

## Abstract

evaluar la efectividad de una intervención educativa en los trabajadores de la limpieza e higienización hospitalaria en la protección de daños causados por agentes biológicos.

estudio prospectivo, casi experimental, del tipo antes y después, con grupo único, con el análisis del comportamiento de los trabajadores en la adhesión al cumplimiento de las precauciones estándar. La recolección de datos se realizó en 106 trabajadores de la limpieza e higienización, por medio de un cuestionario demográfico, ocupacional, de conocimiento y comportamiento sobre la prevención de daños causados por agentes biológicos. Se observó el comportamiento de los participantes relacionados a la bioseguridad, pre y post-intervención educativa. El análisis fue estadístico descriptivo e inferencial.

77,4% de los trabajadores eran del sexo femenino; aproximadamente 42% tenían hasta un año de servicio. En cuanto al grado de escolaridad, 39,6% tenían la Enseñanza Media completa y 29,2% la Enseñanza Fundamental incompleta. La mediana del puntaje de adhesión a las Precauciones Estándar fue: pre intervención 14 puntos y post-intervención 17 puntos. Entre la observación antes de cambiar la ropa por vestimentas adecuadas, la adhesión a la higienización de las manos con agua y jabón fue de 41,7% pasando para 75,0% después de la intervención.

la intervención educativa, fundamentada en talleres teórico-prácticos y metodologías activas, mejoró la adhesión a las prácticas de bioseguridad, lo que fue evidenciado tanto por el comportamiento relatado como por la observación directa. El liderazgo y el compromiso de los gestores hospitalarios son cruciales para la continuidad de la educación y las prácticas de bioseguridad, lo que aumenta la seguridad de los profesionales y pacientes. Estudios futuros deben enfocarse en la salud de los trabajadores de la limpieza e higienización hospitalaria, incluyendo los programas educativos y la relación entre sus actividades y la seguridad de los pacientes contra las infecciones en los servicios de salud.

## Destacados

**(1)** Inclusión de profesionales de higienización y limpieza hospitalaria en las normas de bioseguridad. **(2)** Educación permanente para la promoción de la bioseguridad entre los trabajadores de la limpieza. **(3)** Educación permanente por medio de metodologías. **(4)** La educación permanente promueve la adhesión a los EPIs en trabajadores de la limpieza hospitalaria.

## Introducción

La bioseguridad es definida por las acciones destinadas a la prevención, control, reducción o eliminación de riesgos provenientes de actividades que posibilitan el comprometimiento de la calidad de vida, de la salud humana y del medio ambiente^(1)^. Los riesgos se clasifican por los medios de contaminación en físicos, biológicos, químicos, ergonómicos o de accidente, sea en laboratorios de investigación o en unidades de salud^(2)^.

Los microorganismos patogénicos representan una preocupación significativa en ambientes cerrados, especialmente en servicios como hospitales, debido al riesgo de provocar infecciones hospitalarias^(3)^. Por tanto, los profesionales que actúan en esos ambientes están más susceptibles a las enfermedades infecciosas debido a la exposición frecuente a virus, bacterias y hongos durante el trabajo^(4)^.

Dentro de esa perspectiva, es notable la importancia del uso de Equipamientos de Protección Individual (EPIs) por los funcionarios hospitalarios, considerando la Norma Reglamentaria (NR) nº 6 del Ministerio del Trabajo que promueve el uso de EPI afirmando que “es todo dispositivo o producto, de uso individual, utilizado por el trabajador, destinado a la protección de riesgos para asegurar la salud en el trabajo”^(5)^.

Se destaca que los trabajadores de la limpieza e higienización hospitalaria, diariamente manosean potenciales agentes infectantes que pueden transformarse en un gran núcleo contaminador, en caso de no ser administrados correctamente^(6)^. A pesar del reconocimiento global de la importancia de la limpieza en cuidados de salud, los trabajadores de la limpieza continúan subvalorados y frecuentemente son ignorados por el personal clínico en enfermarías con mucho movimiento^(7)^.

De la misma forma, implementar abordajes de educación en salud a los agentes de limpieza puede perfeccionar su conocimiento sobre la bioseguridad, contribuyendo para un control más eficaz de infecciones en ambientes de salud^(8)^.

Para esto, se destaca la importancia de la Educación Permanente en Salud (EPS) en la construcción del aprendizaje de lo cotidiano del equipo de trabajo. La EPS abarca las actividades educativas en salud, gestión y control social, objetivando analizar críticamente las necesidades de los usuarios y promoviendo los cambios en la realidad del trabajo en salud^(9-9)^. Fortalecer la cultura de seguridad dentro de las instalaciones de salud y suministrar un entrenamiento continuo son componentes esenciales de la EPS; así, contribuyendo para la protección de los trabajadores^(11)^.

Delante de este contexto, es evidente la necesidad de realizar estudios que aborden la EPS en los trabajadores de la limpieza e higienización hospitalaria, con enfoque en bioseguridad. De ese modo, surgen las preguntas orientadoras: “¿Cuál es la efectividad de una intervención educativa, en la adhesión a medidas de bioseguridad por trabajadores de limpieza e higienización hospitalaria, en la protección contra los daños causados por agentes biológicos?” y “¿Las actividades de EPS pueden contribuir para la protección de estos trabajadores de daños causados por agentes biológicos?”.

Las contribuciones de este estudio para el área hospitalaria son múltiples. En primer lugar colocar en evidencia la importancia de intervenciones educativas en la adhesión a las prácticas de bioseguridad; también, el estudio podrá proporcionar un ambiente más seguro, tanto para los profesionales de la salud, como para los pacientes. Además de eso, la formación continua y específica de los trabajadores de la limpieza e higienización podrá reducir la incidencia de infecciones hospitalarias, mejorando la calidad de la atención y los resultados clínicos. Por último, al promover la concientización y el entrenamiento adecuado, se espera que este estudio contribuya para la construcción de una cultura organizacional orientada a la seguridad y prevención de riesgos, fortaleciendo las políticas institucionales de salud y seguridad en el trabajo.

Por tanto, el objetivo de este estudio fue evaluar la efectividad de una intervención educativa en los trabajadores de la limpieza e higienización hospitalaria, referente a la protección contra los daños causados por agentes biológicos.

## Método

### Delineamiento del estudio

Se trata de un estudio prospectivo, casi experimental, del tipo antes y después, de grupo único, con análisis del comportamiento de los trabajadores, en la adhesión al cumplimiento de las precauciones estándar para la protección de daños causados por agentes biológicos. La redacción de este estudio fue orientada por el *checklist* del *Strengthening the Reporting of Observational Studies in Epidemiology* (*Standards for Quality Improvement Reporting Excellence*) (SQUIRE)^(12)^.

### Escenario de la investigación

El estudio fue desarrollado en una unidad de salud de gran porte, localizada en el estado de Tocantins, en Brasil. La institución atiende 100% por el Sistema Único de Salud (SUS), y es caracterizada como hospital de enseñanza, general, público y de nivel terciario. Este local fue seleccionado por tratarse de un hospital escuela y local en donde los equipos de trabajadores de la limpieza e higienización actúan en ambientes con alto riesgo de infección por material biológico. Los sectores que hicieron parte de esta investigación fueron: emergencia adulto, centro quirúrgico e internaciones.

### Población, criterios de selección y definición de la muestra

Todos los trabajadores de la limpieza e higienización hospitalaria actuantes en las unidades de la institución fueron convidados a participar del estudio. Se adoptaron como criterios de inclusión: ser trabajadores de la limpieza e higienización hospitalaria vinculados al hospital investigado, no estar ejerciendo otra función o en cargo de jefatura. Fueron excluidos los que no estaban trabajando por cualquier motivo durante el período de la recolección de datos y los que no participaron de la intervención educativa.

Para las etapas de observación, se utilizó como criterio de elegibilidad a los trabajadores que participaron de las demás etapas de la investigación. Para el cálculo del tamaño del muestreo se consideró la población de 123 trabajadores; se adoptó el intervalo de confianza de 99%, el error de muestreo de 5%, resultando en una muestra mínima de 104 trabajadores. Se consideraron las posibles pérdidas debido a los rechazos y/o los que no estaban trabajando; todos los trabajadores presentes en el ambiente laboral fueron convidados a participar del estudio. En la pre intervención participaron 115, por medio de respuestas a los cuestionarios; en la intervención educativa participaron 106 trabajadores, así como en la post-intervención.

Así, 106 trabajadores participaron de todas las etapas, constituyendo la muestra final compuesta por trabajadores que participaron, tanto de la etapa de pre intervención, como de la intervención y post-intervención.

Para la observación de los investigadores sobre la adhesión a las medidas de bioseguridad en la actividad laboral de los trabajadores de la limpieza e higienización hospitalaria, tanto en la etapa de pre intervención, como en la post-intervención; así fueron observados 12 trabajadores que constituyó la muestra aleatoria simple representando cerca de 10% del total de participantes de la primera etapa de la investigación.

### Instrumentos utilizados

El instrumento de recolección de datos estuvo compuesto por un cuestionario constituido por tres partes: (1) demográfico y ocupacional; (2) conocimiento y comportamiento referido sobre la prevención de daños por agentes biológicos, y (3) indagaciones sobre los tipos de EPI utilizados para las precauciones basadas en la trasmisión.

El cuestionario, demográfico y ocupacional, fue construido por los autores de este estudio, conteniendo los siguientes ítems: sexo biológico, edad, escolaridad, turno de trabajo, tiempo de trabajo y participación previa en capacitación sobre EPI (pre intervención).

El cuestionario de conocimiento y comportamiento, sobre la prevención de daños por agentes biológicos (pre y post-intervención), fue adaptado de la escala de la versión brasileña de la *Compliance With Standard Precautions Scale* (CSPS-PB)^(13)^, validada por medio de procesos metodológicos como: traducción, consenso entre jueces, retrotraducción, validación semántica y pre test.

La escala CSPS representa las alternativas para medir la adhesión de los profesionales de Enfermería en la práctica de control de infecciones. Debido a la falta de instrumentos específicos para los trabajadores de la limpieza e higienización hospitalaria y buscando la adecuación a la actividad laboral de estos, el cuestionario adaptado, a partir del CSPS, fue mantenido con 20 ítems. Los ítems 3, 6, 7, 12, 13, 14, 15, 16 y 17 permanecieron inalterados en relación al original. Los ítems 1, 8, 10 y 19 fueron modificados y los ítems 2, 4, 5, 9, 11, 18 y 20 fueron retirados. Además de eso, fueron adicionados siete nuevos ítems relacionados al uso de máscaras, vestimenta de los EPIs y retirada de la misma. Previamente, el pre test fue realizado con algunos trabajadores de la limpieza e higienización hospitalaria de la institución en donde ocurrió el estudio.

A continuación, los mismos presupuestos de la CSPS-PB, en el cuestionario quedaron con cuatro opciones de respuestas que indican la frecuencia del cumplimiento de la Precaución Estándar (PE), que son: “siempre”, “muchas veces”, “raramente” o “nunca”. Se consideró que los profesionales deberían adherir integralmente a las PE, era esperado que la opción “siempre” estuviese en la mayoría de las respuestas^(13)^.

El puntaje de cumplimiento a las PE varía de cero a 20; cuanto más próximo de 20 mejor es la adhesión de los profesionales. Para la opción de respuesta “siempre”, fue atribuido el puntaje de “uno” y para las opciones “muchas veces”, “raramente” o “nunca” el puntaje atribuido fue “cero”.

Para registrar la observación del comportamiento de los participantes (pre y post-intervención) se utilizó un formulario denominado *Checklist* de evaluación de la adhesión de las medidas de prevención de daños por agente biológicos (utilización de EPIs). Este instrumento es una versión adaptada del *Checklist* de los EPIs para la vestimenta de trabajo y retirada de la misma^(14-14)^.

### Recolección de datos

La recolección de datos sucedió en el período de diciembre de 2022 a abril de 2023, fue realizada por investigadores previamente capacitados, realizada en cuatro etapas, distribuidas durante la pre intervención, intervención y post-intervención.

En la primera etapa se realizó una reunión con los trabajadores de cada turno de trabajo para la presentación del estudio, invitación y aplicación del cuestionario sobre el perfil demográfico, ocupacional, de conocimiento y comportamiento declarado, en relación a la prevención de daños por agentes biológicos.

Los cuestionarios respondidos fueron colocados por el propio participante en un sobre sin identificación, lacrado y entregado a los investigadores al término del llenado. Durante la recolección de datos, los investigadores quedaron disponibles para eventuales dudas en el llenado del cuestionario.

La segunda etapa comprendió la fase de observación del comportamiento de los participantes (pre y post-intervención) para la evaluación de la adhesión de las medidas de prevención de daños por agentes biológicos (utilización de EPIs). Se realizó en seis días de campo, totalizando 72 horas, siendo posible realizarlas en todos los turnos.

Fueron observadas las diferentes dinámicas y rutinas del servicio, en horarios y períodos alternados, considerando las posibilidades presentadas por el campo. Esa aproximación fue fundamental para la clareza, identificación y comprensión de la organización y de la dinámica del proceso de vestimenta de trabajo y retirada de la misma en el escenario estudiado.

Los observadores evitaron establecer contacto directo con los participantes para no influenciar el comportamiento de los mismos. En la observación de los participantes y del registro de los comportamientos, cada trabajador fue observado durante momentos alternados, por dos investigadores^(16)^.

En la tercera etapa de la recolección de datos, fue implementada la intervención educativa. Para esto, se elaboró un plan de enseñanza de acuerdo con la necesidad de educación permanente, identificado por medio de los cuestionarios aplicados y por la observación de los participantes.

Esta etapa fue realizada en el formato de taller teórico práctico, con la utilización de metodologías activas, conteniendo las siguientes temáticas: técnica de higienización de las manos; riesgos biológicos y vías de contaminación; secuencia indicada para la vestimenta y su retirada; tipos de precauciones; y, EPI conforme al tipo de precaución.

Como parte de las metodologías activas se utilizó la Práctica de Higienización de las manos en la “Caja de la Verdad”; Teatro “Simulación del riesgo de contaminación”, en el cual se aclaró la transmisibilidad de agentes biológicos; Taller de vestimenta y su retirada y “Juego de los Errores”, por medio de imágenes y simulaciones de situaciones de trabajo en el cual el trabajador apuntaba los errores relativos a la utilización de EPIs.

En la cuarta etapa, después un mes de la realización de la intervención educativa, fue efectuado el post test aplicando el cuestionario conocimiento y comportamiento referido a la prevención de daños por agentes biológicos y observación de los participantes post-intervención.

### Intervención educativa

La intervención educativa con los trabajadores de limpieza e higienización hospitalaria fue desarrollada después de identificar el conocimiento previo y observación de las prácticas relacionadas a la bioseguridad.

Se optó por el formato de talleres teórico prácticos, trabajando momentos dinámicos para la asimilación de los contenidos explicados. Las actividades realizadas en esas dinámicas fueron:

*Simulación de la higienización de las manos en la “Caja de la Verdad”* - Consiste en una caja organizadora con una abertura lateral para la inserción de las manos y una abertura en la tapa para visualización. En el lateral interno derecho, se incorporó una luz negra de cinco watts. Para realzar la iluminación proveniente de la luz negra, el interior de la caja fue revestido con color negro.

Como método de higienización, se adoptó una solución de alcohol en gelatinoso a 70%, fue adicionada con una pintura transparente que se tornaría fluorescente en la presencia de la luz negra. Esa solución fue puesta a disposición de los profesionales para la higienización de las manos.

Después de la aplicación, los profesionales fueron conducidos hasta la “Caja de la Verdad” para verificar la cobertura de las manos con la solución alcohólica fluorescente, identificando la presencia de áreas no higienizadas adecuadamente.

*Pieza teatral* - Durante la demonstración realizada por el miembro del equipo de investigación, fue presentada la simulación de la trasmisión de agentes biológicos. La escena retrató al profesional recogiendo basura contaminada, con el saco de basura previamente sucio con pintura guache para representar los agentes biológicos.

En la escenificación, después de recolectar la basura, el profesional toca una perilla de una puerta y luego toca su máscara, rascando la nariz con los guantes que manipularon el saco de basura contaminado. Posteriormente, al remover los guantes, el profesional omite la higienización de las manos, saluda a su compañero de trabajo con un apretón de manos y continúa sus actividades laborales.

*Seguridad del trabajo -* Esta dinámica envuelve el uso de dos globos, inflados con aire, siendo que apenas uno de ellos fue envuelto por cinta adhesiva transparente. Fue explicado que el globo sin cinta representaba el profesional que no utiliza los EPIs o que lo hace de manera inadecuada, en cuanto el globo con cinta representaba los profesionales que utilizan los EPIs correctamente.

Una aguja simbolizó el accidente de trabajo y los riesgos de contaminación. Al introducir la aguja en el globo sin cinta, este se rompe fácilmente, en cuanto al inserirla en el globo con la cinta, el globo permaneció intacto.

*Juego de los errores* - Fueron exhibidas imágenes o simulaciones de situaciones de trabajo que evidenciaban los equívocos en el uso de EPIs, presentando los riesgos de contaminación, así como las inadecuaciones en las prácticas de vestimenta de trabajo y de su retirada. Los trabajadores identificaron los errores en las escenas y apuntaron las acciones correctas apropiadas para cada situación.

¿Qué *está faltando?* – Fueron presentados actores (investigadores) vestidos con EPIs, conforme las indicaciones de todas las precauciones. Los trabajadores, divididos en grupos, observaron e identificaron, verbalmente, cuáles EPIs estaban faltando.

Así, el taller pedagógico fue concebido con fundamentos en el aprendizaje significativo, abordaje multimodal y aplicación de metodologías activas. Estos incluyeron discusiones sobre los temas presentados, así como los momentos dedicados a las actividades prácticas, objetivando proporcionar una comprensión más profunda para los trabajadores en el área de limpieza e higienización hospitalaria.

## Análisis de los datos

El análisis descriptivo utilizó frecuencias absolutas y relativas para las variables cualitativas, y por medias y desviación-estándar para las cuantitativas. El test de McNemar fue utilizado para comparar los ítems que componen la PE en los momentos pre y post-intervención; el puntaje obtenido de la adhesión fue comparado por el test de Wilcoxon, por no haber sido observada la adherencia de los datos a la distribución normal, que a su vez fue evaluada por el test de Shapiro-Wilk. El nivel de significación fue de 5%. El programa utilizado fue el *Stata* (*StataCorp*, LC) versión 15.0.

Los registros de la observación realizados por los dos investigadores tuvieron como grado de concordancia el coeficiente *kappa* 0,94, que evidenció una alta concordancia.

### Aspectos éticos

Este estudio fue realizado de conformidad con la Resolución nº 466/12 del Consejo Nacional de Salud (CNS)^(17)^. El proyecto obtuvo la autorización institucional para la recolección de datos y fue apreciado y aprobado por el Comité de Ética en Investigación (CEI) de la Universidad Federal de Tocantins (UFT) (CAAE-63028922.2.0000.5519/Parecer 5.694.479).

## Resultados

La edad media de los participantes del estudio fue de 40,9 años (DE 11,9) siendo la edad mínima 21 años y la máxima 67 años. La Tabla 1 presenta el perfil demográfico y ocupacional de los 106 trabajadores de la limpieza e higienización hospitalaria.

**Tabla 1 -:** Distribución numérica y porcentaje del perfil demográfico y ocupacional de los trabajadores de la limpieza e higienización hospitalaria (n = 106). Tocantins, TO, Brasil, 2022-2023

Variables	n (%)
**Sexo biológico**	
Femenino	82 (77,4)
Masculino	24 (22,6)
**Escolaridad**	
Fundamental completa	10 (9,4)
Fundamental incompleta	31 (29,2)
Media completa	42 (39,6)
Media incompleta	21 (19,8)
Superior completa	1 (0,9)
Superior incompleta	1 (0,9)
**Turno de trabajo**	
Diurno	63 (59,4)
Nocturno	43 (40,6)
**Tiempo de trabajo (años)**	
Hasta 1	40 (37,8)
1 a < 2	27 (25,4)
2 a < 3	7 (6,6)
3 o más	32 (30,2)
Participación anterior en capacitaciones sobre EPI*	
Si	95 (89,6)
No	11 (10,4)
**EPI Teórica/Práctica**	
Teórica	75 (70,7)
Práctica	26 (24,6)
Ambas	5 (4,7)
Cuántas veces participó de capacitaciones sobre EPI*	
01-2	63 (59,4)
03-4	(19,9)
5+	22 (20,7)

*Equipamiento de Protección Individual

Dentro del espectro del sexo biológico, se evidenció una marcada predominancia femenina con 77,4%. En relación a la formación educacional, cerca de 40% de los profesionales terminaron la Enseñanza Media y cerca de un tercio no concluyó la Enseñanza Fundamental. Una mera fracción, 1,8%, ascendió a la Enseñanza Superior. Notablemente, 59,4% de los entrevistados actuaban en el turno diurno.

Al considerar el tiempo de trabajo, aproximadamente 42% tenían hasta un año de servicio; un cuarto situándose entre uno y dos años de actividad. El resto se distribuía entre dos y tres años o ultrapasaba el marco de tres años.

En el centro de la capacitación profesional se destaca la formación en EPIs. En relación a esta variable 89,6% ya participó de entrenamientos anteriores. Entre tanto, se identificó que 70,7% son de naturaleza teórica, a pesar de que cerca de un cuarto tuvieron una vertiente práctica o teórico práctica.

La Tabla 2 ilustra el porcentaje de adhesión pre y post-intervención educativa para cada ítem del cuestionario de adhesión a las precauciones estándar.

**Tabla 2 -:** Adhesión al cumplimiento de las precauciones estándar en los momentos pre y post-intervención por trabajadores de la limpieza e higienización hospitalaria (n = 106). Tocantins, TO, Brasil, 2022-2023

**Cuestiones**	**Pre intervención**	**Post intervención**	p*
1. Yo lavo mis manos cuando toco en puertas y equipamientos contaminados.	72 (67,9%)	106 (100,0%)	<0,001
2. Yo uso producto a base de alcohol para higienizar las manos como una alternativa, en caso de no estar visiblemente sucias.	69 (65,1%)	96 (90,6%)	<0,001
3. La caja de materiales punzo cortantes es descartada solo cuando está llena.	96 (90,6%)	106 (100,0%)	0,002
4. Yo retiro los EPIs ^†^ en un local designado.	59 (55,7%)	86 (81,1%)	<0,001
5. Yo tomo baño y cambio de ropa privativa en caso de grandes respingos, inclusive si yo hubiese usado EPI ^†^ .	63 (59,4%)	83 (78,3%)	0,002
6. Yo uso guantes largos de goma cuando estoy expuesta a los fluidos corporales, a la sangre o a los derivados y a cualquier excreción de pacientes.	52 (49,1%)	0 (0%)	<0,001
7. Yo higienizo mis manos inmediatamente después remover los guantes.	92 (86,8%)	106 (100,0%)	<0,001
8. Yo uso máscara quirúrgica en combinación con anteojos de protección y delantal, siempre que existe la posibilidad de salpicaduras o derramamientos.	40 (37,7%)	75 (70,8%)	<0,001
9. Mi boca y mi nariz quedan cubiertos cuando uso máscara.	95 (89,6%)	106 (100,0%)	0,001
10. Yo reutilizo una máscara quirúrgica o EPI ^†^ descartable.	71 (67,0%)	106 (100,0%)	<0,001
11. Yo uso delantal/capote cuando estoy expuesto a la sangre, a fluidos corporales o a cualquier excreción de pacientes.	68 (64,2%)	106 (100,0%)	<0,001
12. Yo descarto material contaminado con sangre, fluidos corporales, secreciones y excreciones de pacientes, en sacos plásticos blancos, independientemente del estado infeccioso del paciente.	98 (92,5%)	106 (100,0%)	0,008
13. Yo uso guantes de procedimiento para descontaminar equipamientos que presenta suciedad visible.	94 (88,7%)	106 (100,0%)	<0,001
14. Yo evito tocar ojos, nariz y boca con las manos no lavadas/higienizadas.	93 (87,7%)	106 (100,0%)	<0,001
15. Yo evito tocar superficies (ej.: muebles, puertas y equipamientos para la salud) con guantes u otros EPIs ^†^ contaminados o con las manos contaminadas.	78 (73,6%)	106 (100,0%)	<0,001
16. Yo evito tocar en la parte de la frente de la máscara y cuando eso ocurre, inmediatamente higienizo las manos.	67 (63,2%)	106 (100,0%)	<0,001
17. Yo remuevo la máscara usando la técnica apropiada (o sea, no toco en la frente de la máscara, pero la remuevo por las tiras).	74 (69,8%)	106 (100,0%)	<0,001
18 Yo realizo la higienización de las manos después de la remoción de la máscara.	86 (81,1%)	106 (100,0%)	<0,001
19. Después de utilizar yo descarto los EPIs ^†^ descartables en la basura para residuo infectante (saco blanco).	86 (81,1%)	106 (100,0%)	<0,001
20. Después de utilizar, yo retiro el delantal tratando de sujetarlo por la parte interna del mismo, evitando contaminarme.	75 (70,8%)	106 (100,0%)	<0,001

*McNemar p = Valor de *p* para comparación entre pre y post-intervención educativa; ^†^Equipamiento de Protección Individual

Inicialmente, se destaca que en la comparación del comportamiento referido antes y después de la intervención educativa, todos los ítems presentaron positivamente una diferencia estadísticamente significativa, con valor de p< 0,001. En contraste, el ítem 7 - “uso guantes de goma cuando estoy expuesto a fluidos corporales, sangre o derivados y a cualquier excreción de pacientes” - pasó a ser cero después de la intervención, debido a la ausencia del EPI en el servicio (substituido por guante de látex).

La mediana del puntaje global de adhesión a las PE, comparada entre pre y post-intervención, fue mayor en la post-intervención con 17 puntos (Figura 1).

La Tabla 3 presenta la adhesión a los EPIs específicos para las precauciones respiratorias antes y después de la intervención.

**Figura 1 -:** Efectos de la intervención educativa sobre la puntuación de la adhesión a las precauciones estándar de los trabajadores de la limpieza e higienización hospitalaria (n = 106). Tocantins, TO, Brasil, 2023

**Tabla 3 -:** Adhesión a los EPIs* para la precaución específica respiratoria en los momentos pre y post-intervención de los trabajadores de la limpieza e higienización hospitalaria (n = 106). Tocantins, TO, Brasil, 2023

Variables	Pre				Post			
	**Siempre**	**A veces**	**Raramente**	**Nunca**	**Siempre**	**A veces**	**Raramente**	**Nunca**
***Transmisión respiratoria- aerosoles***								
Gorro	99 (93,4%)	3 (2,8%)		4 (3,8%)	106 (100,0%)			
Máscara quirúrgica	24 (22,6%)	1 (0,9%)	4 (3,8%)	77 (72,7%)				106 (100,0%)
Delantal de mangas largas (no impermeable)	80 (75,5%)	10 (9,4%)	7 (6,6%)	9 (8,5%)	106 (100,0%)			
Guantes de procedimiento	93 (87,8%)	7 (6,6%)	1 (0,9%)	5 (4,7%)	106 (100,0%)			
Máscara N95 o PFF2 ^†^	88 (83,0%)	14 (13,2%)		4 (3,8%)	106 (100,0%)			
Anteojos de protección o protector facial	31 (29,2%)	13 (12,3%)	9 (8,5%)	53 (50,0%)				106 (100,0%)
Delantal de mangas largas impermeable	33 (31,1%)	11 (10,4%)	9 (8,5%)	53 (50,0%)				106 (100,0%)
Test de sellado de la N95, PFF2 ^†^ o similar	65 (61,3%)	12 (11,3%)	8 (7,6%)	21 (19,8%)	84 (79,3%)	15 (14,1%)	2 (1,9%)	5 (4,7%)
***Trasmisión respiratoria-gotículas***								
Máscara quirúrgica	80 (75,5%)	6 (5,7%)	2 (1,8%)	18 (17,0%)	106 (100,0%)			
Delantal de mangas largas (no impermeable)	80 (75,5%)	15 (14,2%)	1 (0,9%)	10 (9,4%)	106 (100,0%)			
Guantes de procedimiento	93 (87,7%)	9 (8,5%)	1 (0,9%)	3 (2,9%)	106 (100,0%)			
Anteojos de protección o protector facial	32 (30,2%)	13 (12,3%)	8 (7,5%)	53 (50,0%)				106 (100,0%)
Delantal de mangas largas impermeable	36 (34,0%)	6 (5,7%)	8 (7,5%)	56 (52,8%)				106 (100,0%)
Delantal de paño o lona estándar	19 (17,9%)	9 (8,5%)	8 (7,6%)	70 (66,0%)				106 (100,0%)

*Equipamiento de Protección Individual; ^†^Pieza Facial Filtrante Clase 2

Por el análisis descriptivo simple, se observó un aumento en el porcentaje de adhesión a los diversos EPIs, tanto para las precauciones para los aerosoles, como para las gotículas, con destaque para ítems como gorro, delantal de mangas largas (no impermeable), guantes de procedimiento, y máscara N95 o Pieza Facial Filtrante Clase 2 (PFF2), la adhesión pasó a ser de 100% (“siempre”).

En el ámbito de la trasmisión respiratoria por aerosoles, la adhesión al uso continuo de gorros, delantales de mangas largas (no impermeables), guantes de procedimiento, máscaras N95 o PFF2 después de la intervención fue de 100%. Antes de la intervención, la utilización de máscaras quirúrgicas era baja, con apenas 22,6% de los profesionales usándolas siempre, y 72,6% nunca utilizándolas. Sin embargo, en el período post-intervención, el uso de ese EPI cayó para cero, siendo ese, el resultado esperado, ya que el protocolo es no utilizar máscaras quirúrgicas en precauciones de trasmisión respiratoria por aerosoles.

En relación al test de sellado de la N95, PFF2 o similares, se nota que hubo una elevación en la adhesión de 61,3% en el período pre intervención para 79,3% en la post-intervención.

En relación a la trasmisión respiratoria por gotículas, se observa que 75,5% de los profesionales siempre usaban máscaras quirúrgicas y delantales de mangas largas (no impermeables) en el momento de la pre intervención.

En la post-intervención, esa tasa alcanzó 100%. Los guantes de procedimiento eran frecuentemente usados por 87,7% de los entrevistados antes de la intervención, valor que posteriormente también subió a 100%. Por otro lado, antes de la intervención 30,2% de los profesionales siempre utilizaban anteojos de protección o protector facial y 34,0%, usaban constantemente el delantal impermeable de mangas largas.

Después de la intervención, las dos prácticas fueron completamente descontinuadas. El delantal estándar de paño o lona tenía una adhesión de apenas 17,9% en la pre intervención, y tal como los otros EPIs mencionados, su uso también cesó en la post-intervención.

La Tabla 4 presenta los ítems observados por los investigadores en actos anteriores al vestimento de la ropa de trabajo, durante la misma y en su retirada, realizados por los trabajadores de la limpieza e higienización, cuando de forma aleatoria fueron observados los 12 profesionales antes y después de la intervención, en relación a las medidas de bioseguridad.

**Tabla 4 -:** Procedimientos observados antes y durante la colocación de la indumentaria de trabajo y la retirada de la misma, para comprobar las precauciones estándar en los momentos pre y post-intervención educativa de los trabajadores de la limpieza e higienización hospitalaria (n = 12). Tocantins, TO, 2022-2023

Antes de vestir la indumentaria	Pre intervención	Post intervención
Higieniza las manos con agua y jabón	5 (41,7%)	9 (75,0%)
Separa y valida la integridad de los EPI*	11 (91,7%)	12 (100,0%)
***Delantal:*** prende los lazos del cuello y de la cintura	7 (58,3%)	12 (100,0%)
**Durante la colocación de la indumentaria**	Pre intervención	Post intervención
***Máscara quirúrgica***		
Usa máscara quirúrgica	11 (91,7%)	12 (100,0%)
Posiciona el clip nasal sobre el dorso de la nariz	11 (91,7%)	12 (100,0%)
Posiciona los elásticos atrás de las orejas o amarra las cintas	11 (91,7%)	12 (100,0%)
Confirma que la máscara cobre la nariz, boca y mentón	7 (58,3%)	10 (83,3%)
***Anteojos***		
No usa	12 (100,0%)	12 (100,0%)
***Protector facial***		
No usa	12 (100,0%)	12 (100,0%)
***Calza los guantes de procedimiento*** cubriendo los puños de las mangas del delantal	12 (100,0%)	12 (100,0%)
Coloca el ***Gorro*** cubriendo los cabellos y orejas	11 (91,7%)	12 (100,0%)
***Bota larga de goma***		
No usa	12 (100,0%)	12 (100,0%)
**Durante la retirada de la vestimenta**		
***Guantes de procedimiento***		
Sujeta en el puño del guante, de la mano no dominante, y retira el primer guante	8 (66,7%)	12 (100,0%)
Posiciona el dedo indicador bajo el puño del guante y retira el segundo guante	1 (8,3%)	12 (100,0%)
No	11 (91,7%)	0 (0,0%)
Descarta las guantes en la basura infectante	12 (100,0%)	12 (100,0%)
***Higieniza las manos*** con preparación alcohólica a 70%	6 (50,0%)	12 (100,0%)
***Delantal***		
Remueve primero el lazo del cuello y después de la cintura	5 (41,7%)	11 (91,7%)
Posiciona el dedo indicador en la parte interna del puño y tira el delantal cubriendo la mano no dominante	2 (16,7%)	8 (66,7%)
Con la mano protegida por la manga del delantal, sujeta la otra manga, en cuanto desliza y retira el brazo y la mano dominante del delantal	1 (8,3%)	10 (83,3%)
Sujeta la parte interna del delantal (próximo al hombro) y retira la manga del brazo no dominante	1 (8,3%)	10 (83,3%)
Continua virando el delantal de dentro hacia fuera, doblando y formando un paquete	1 (8,3%)	10 (83,3%)
Descarta el delantal en la basura infectante	9 (75,0%)	12 (100,0%)
***Higieniza las manos*** con preparación alcohólica a 70%	6 (50,0%)	12 (100,0%)
***Anteojos o protector***		
No usa	12 (100,0%)	12 (100,0%)
***Gorro-estaba usando***	11 (91,7%)	12 (100,0%)
Posiciona los dedos indicadores dentro del gorro al lado de las orejas y desliza el gorro para arriba, para atrás y para el lado	8 (66,7%)	12 (100,0%)
Descarta el gorro en la basura infectante	11 (91,7%)	12 (100,0%)
***Higieniza las manos*** con preparación alcohólica a 70%	1 (8,3%)	10 (83,3%)
***Máscara quirúrgica***		
Estaba usando	9 (75,0%)	12 (100,0%)
Sujeta los elásticos atrás de las orejas, o deshace los lazos de las cintas en la región posterior de la cabeza	9 (75,0%)	12 (100,0%)
Tira la máscara hacia adelante, sujetando los elásticos o las cintas, sin tocar en su superficie	8 (66,6%)	12 (100,0%)
Descarta la máscara en la basura infectante	9 (75,0%)	12 (100,0%)
Higieniza las manos con preparación alcohólica a 70%	0 (0,0%)	9 (75,0%)
***Bota larga de goma impermeable***		
No estaba usando	12 (100,0%)	12 (100,0%)

*Equipamiento de Protección Individual

Se observa que todos los ítems evaluados “antes de colocar la indumentaria” tuvieron aumento de adhesión post-intervención educativa.

Durante la colocación de la indumentaria, todos los procedimientos asociados a la máscara quirúrgica, incluyendo su uso, posicionamiento y ajuste, exhibieron una adhesión de 100% después de la intervención, con excepción del procedimiento de garantizar que la máscara cubriese la nariz, boca y mentón, que aumentó de 58,3% para 83,3%. En contraste, el uso del gorro, que cubre cabellos y orejas alcanzó 100% de adhesión después de la intervención.

En relación a los procedimientos observados durante la retirada de la indumentaria, hubo un aumento de la adhesión en muchas prácticas. En el caso de la retirada de los guantes, la técnica de sujetar el puño del guante de la mano no dominante para retirar el primer guante, y la de posicionar el dedo indicador bajo el puño del guante para retirar el segundo, fue observado que ambos alcanzaron 100% de adhesión después de la intervención.

La higienización de las manos con preparación alcohólica a 70% después de la retirada de la indumentaria (delantal y la máscara quirúrgica) pasó de 50,0% y 0,0% para 100,0% y 75,0%, respectivamente. El procedimiento de remover el delantal, virándolo de adentro hacia afuera y doblándolo, aumentó de 8,3% para 83,3%.

## Discusión

Este estudio tuvo como enfoque el análisis del perfil demográfico y ocupacional de los trabajadores de la limpieza e higienización hospitalaria; fue realizado en un hospital en el estado de Tocantins, Brasil, en 2022/2023; también se estudió la efectividad de la intervención de educación permanente sobre bioseguridad.

En consonancia con estudios anteriores, se identificó una predominancia femenina en la limpieza e higienización hospitalaria^(18)^.

En relación a la formación educacional, un análisis minucioso reveló matices críticos; la limitada presencia de profesionales con educación superior surgió con la literatura, lo que sugiere que esas funciones son comúnmente ocupadas por individuos con menos años de formación académica^(19)^.

La distribución equitativa entre los turnos de trabajo diurno y nocturno puede afectar tanto el bienestar de los profesionales, como la eficacia operacional de las instituciones. Los hospitales funcionan 24 horas por día, exigiendo que los profesionales de la limpieza estén preparados para actuar en turnos que cubran todo el período (diurno y nocturno). La manutención de la higiene hospitalaria es esencial a lo largo del día; sin embargo, el trabajo nocturno puede presentar desafíos adicionales para la salud^(20)^. Los turnos de trabajo para el personal con edad avanzada o sin experiencia, deben ser organizados en períodos cortos durante la tarde, objetivando minimizar el riesgo de accidentes de trabajo. Además de eso, el personal con edad avanzada debe ser asignado en unidades de bajo riesgo, para así prevenir accidentes^(21)^.

Los datos destacaron una alta participación en capacitaciones anteriores sobre EPIs, siendo la mayor parte de naturaleza teórica. Esa dicotomía en el entrenamiento, concomitantemente con la frecuencia de actualizaciones de EPI, conduce a reflexionar sobre la efectividad y calidad del entrenamiento ofrecido. La carencia de entrenamiento adecuado también fue percibida en otros departamentos, en los cuales los participantes declararon no disponer de todas las informaciones necesarias para desempeñar sus tareas^(22)^.

Al reforzar la importancia de implementar entrenamientos eficaces, en las unidades de salud a partir de las necesidades de los trabajadores, este abordaje proporciona más chances de aprendizaje, resultando en una adhesión de los profesionales más amplia a las medidas de protección individual y al mismo tiempo reduce la vulnerabilidad a los riesgos^(23)^. Por otro lado, el conocimiento por sí solo, no indica necesariamente un cambio de comportamiento, ya que este puede estar relacionado a varios factores intrínsecos y extrínsecos.

En ese sentido, la calificación en salud está vinculada a la adquisición de conocimiento y transformación de comportamientos individuales; esto puede ser conceptuado como un conjunto de competencias fundamentales y recursos contextuales que las personas necesitan adquirir para comprender, evaluar, comunicar y aplicar las informaciones y prestar los servicios de diversas maneras, en escenarios diferentes, a lo largo de sus vidas, con el propósito de mejorar su salud y bienestar^(24)^.

La educación en salud tiene el potencial de promover cambios en los comportamientos humanos; esta puede incentivar la reflexión sobre la organización del trabajo de limpieza, identificar los factores que causan enfermedades y discutir las estrategias para reducirlos. Esto ayuda a dar un nuevo significado a las prácticas de autocuidado y a mejorar la capacidad de enfrentar los problemas que afectan la salud física y mental de los trabajadores del Servicio Hospitalario de Limpieza^(25)^.

De esa forma, las competencias que van más allá del simple conocimiento y entrenamiento, desempeñan un papel significativo en el desarrollo de habilidades prácticas; por ejemplo, el vestir la indumentaria y su retirada, la higienización de las manos; así como, influyen en la formación de aptitudes positivas. Lo anterior destaca el impacto benéfico de la intervención realizada por medio de talleres teórico-prácticos que emplean metodologías activas.

Después de la intervención educativa, se observó un aumento expresivo de la adhesión a las precauciones estándar. Estos resultados están en sintonía con la literatura existente, que identifica la efectividad de varias estrategias, incluyendo el entrenamiento, el *feedback* y los sistemas facilitadores^(26-26)^.

Fue percibido un aumento significativo de la utilización de alcohol como agente de higiene (cuando las manos no presentaban suciedad visible), evidenciado un vacío en el conocimiento combinado con la falta de la práctica diaria. La higienización de las manos - sea con agua y jabón líquido o preparaciones alcohólicas, en conformidad con los cinco momentos establecidos para esa práctica - en los servicios de salud, debe ser una rutina previamente internalizada por todos los profesionales de la salud. Es imperativo reiterar su importancia como una medida fundamental para la prevención y el control de infecciones, tanto en el presente, como en el futuro^(28)^. Por tanto, es crucial que las unidades de salud ofrezcan entrenamientos continuos, objetivando obtener la conformidad con los estándares de higiene de las manos, establecidos por los órganos competentes^(29-29)^.

La conexión entre la implementación de prácticas de bioseguridad, la preservación de la salud de los trabajadores y la seguridad de los pacientes es vital, lo que enfatiza que la bioseguridad es un procedimiento funcional y operacional muy relevante, en los diversos servicios de salud, que debe ser reconocido como un instrumento de protección, tanto para el paciente, como para los profesionales que prestan cuidados de salud^(31)^.

Hubo mejorías notables en los siguientes aspectos: identificación del local correcto para retirar los EPIs; uso adecuado de los EPIs al lidiar con fluidos corporales; comprensión de las combinaciones ideales de EPIs para garantizar la seguridad, tanto del profesional, como del paciente; consideraciones sobre la reutilización de los EPIs; utilización de los EPIs para la descontaminación de equipamientos y superficies; y, adopción correcta de medidas para evitar la contaminación durante el proceso de retirada de la indumentaria. Esta mejoría está alineada con estudios anteriores que enfatizan la protección proporcionada por esos equipamientos en ambientes hospitalarios^(32)^.

Conforme las directrices de la Agencia Nacional de Vigilancia Sanitaria de Brasil (ANVISA), se recomienda que los profesionales de higiene y limpieza, en servicios hospitalarios utilicen: anteojos o protector facial (especialmente cuando existe riesgo de salpicaduras de materiales orgánicos o químicos) y delantal impermeable con guantes largos de goma y botas largas impermeables (si existe riesgo de contacto con fluidos o secreciones del paciente que puedan ultrapasar la barrera del delantal de contacto)^(28)^.

En cuanto al descarte de EPIs y materiales hospitalarios, todos los profesionales demostraron plena adhesión a los ítems 3, 12 y 19 en el cuestionario de conocimiento auto declarado; esto es corroborado por otros estudios, en los cuales el descarte impropio de materiales fue identificado como una de las principales causas de accidentes con material biológico, lo que ocurre con mayor frecuencia cuando los profesionales no utilizan EPIs, representando 51% de los casos^(33)^.

La adhesión a algunas prácticas de EPI antes de la intervención era modesta. Esta baja adhesión pre-intervención sugirió la existencia de falta de sensibilidad o de comprensión de la importancia de los EPIs. En proceso de retirar de la indumentaria, hubo un aumento considerable en la adhesión a los procedimientos correctos post-intervención, alineándose con las directrices que destacan la importancia de este proceso para evitar las contaminaciones^(34)^.

El estudio se justifica por la escasez de trabajos científicos enfocados en la población de los trabajadores de limpieza e higienización hospitalaria. Entre las limitaciones encontradas se destaca la falta de cuestionarios validados, que aborden las medidas de bioseguridad específicas para esos trabajadores. El cuestionario de precaución estándar no menciona los EPIs apropiados para esa categoría, como guantes largos de goma y botas largas de goma impermeable. Por tanto, se sugiere la realización de otros estudios para validar los cuestionarios de bioseguridad orientados para esa población.

Otra limitación del estudio es la aplicación de los resultados obtenidos. La observación de los participantes puede interferir en su comportamiento, en relación a la adhesión a las medidas de bioseguridad, ya que pueden percibir que están siendo observados en aquel momento, lo que puede alterar sus acciones.

Las implicaciones para el profesional son significativas, ya que el estudio destaca la necesidad de desarrollar y validar herramientas específicas para evaluar la adhesión a las prácticas de bioseguridad, entre los trabajadores de limpieza e higienización hospitalaria. Además de eso, refuerza la importancia de contar con estrategias de formación y concientización continuas para garantizar la seguridad y salud de esos profesionales y evitar la contaminación en el ambiente hospitalario.

## Conclusión

Hubo una notable mejoría en la adhesión a las precauciones estándar, detectado en los comportamientos relatados, que incluyen el uso adecuado de EPI, higienización de las manos, descarte apropiado de materiales y medidas de prevención de la contaminación del ambiente, lo que fue evidenciado estadísticamente por el aumento en el puntaje de adhesión a las PE.

Por medio del análisis descriptivo, al comparar el porcentaje de utilización de EPIs en precauciones específicas respiratorias, fue observado que la adhesión alcanzó consistentemente 100% para los ítems como gorro, delantal de mangas largas (no impermeable), guantes de procedimiento y máscara N95 o PFF2.

También merece destaque la evaluación de los resultados del Comportamiento Observado, con énfasis en los cambios de comportamiento, como: la correcta higienización de las manos; el uso apropiado del delantal durante el proceso de vestir la indumentaria (y de su retirada); la garantía de que la máscara cubra adecuadamente la nariz, boca y mentón; la forma adecuada de vestir la indumentaria y su retirada (también de los guantes de procedimiento), en relación a la adhesión antes y durante el proceso de vestir la indumentaria (y su retirada), para cumplir con las precauciones estándar.

Así, fue posible verificar que la intervención educativa realizada por medio de taller teórico-práctico, con la utilización de metodologías activas, a partir de las necesidades de los trabajadores, proporcionó una mejora en la adhesión a las medidas de bioseguridad evidenciadas, tanto por el comportamiento auto informado, como por la observación directa.

Se concluye que el uso de la intervención educativa con las orientaciones de bioseguridad, dirigida a los trabajadores de la limpieza e higienización hospitalaria, fue efectiva y contribuyó para alcanzar mayores porcentajes de conocimiento y obtener cambios de comportamientos individuales y colectivos.

Además, el papel de los gestores hospitalarios es crucial en la elaboración e implementación de estrategias que apoyen el desarrollo continuo de los trabajadores de la limpieza e higiene. El liderazgo efectivo y el comprometimiento de los gestores son fundamentales para obtener la continuidad de la educación y el cumplimiento de las prácticas de bioseguridad, garantizando, no apenas la seguridad de los profesionales, pero también la protección de los pacientes contra las infecciones en los servicios de salud.

Se sugiere la realización de estudios posteriores que enfoquen la salud de los profesionales responsables por la limpieza e higienización hospitalaria, incluyendo la implementación de programas educativos orientados a ese grupo. Además de eso, es relevante establecer conexiones entre las actividades desempeñadas por esos profesionales y la seguridad de los pacientes en relación a las infecciones en los servicios de salud.
